# *Streptomyces antioxidans* sp. nov., a Novel Mangrove Soil Actinobacterium with Antioxidative and Neuroprotective Potentials

**DOI:** 10.3389/fmicb.2016.00899

**Published:** 2016-06-16

**Authors:** Hooi-Leng Ser, Loh Teng-Hern Tan, Uma D. Palanisamy, Sri N. Abd Malek, Wai-Fong Yin, Kok-Gan Chan, Bey-Hing Goh, Learn-Han Lee

**Affiliations:** ^1^Novel Bacteria and Drug Discovery Research Group, School of Pharmacy, Monash University MalaysiaBandar Sunway, Malaysia; ^2^Biomedical Research Laboratory, Jeffrey Cheah School of Medicine and Health Sciences, Monash University MalaysiaBandar Sunway, Malaysia; ^3^Biochemistry Program, Faculty of Science, Institute of Biological Sciences, University of MalayaKuala Lumpur, Malaysia; ^4^Division of Genetics and Molecular Biology, Faculty of Science, Institute of Biological Sciences, University of MalayaKuala Lumpur, Malaysia; ^5^Center of Health Outcomes Research and Therapeutic Safety (Cohorts), School of Pharmaceutical Sciences, University of PhayaoPhayao, Thailand

**Keywords:** *Streptomyces antioxidans*, actinobacteria, mangrove, neuroprotective, antioxidative

## Abstract

A novel strain, *Streptomyces antioxidans* MUSC 164^T^ was recovered from mangrove forest soil located at Tanjung Lumpur, Malaysia. The Gram-positive bacterium forms yellowish-white aerial and brilliant greenish yellow substrate mycelium on ISP 2 agar. A polyphasic approach was used to determine the taxonomy status of strain MUSC 164^T^. The strain showed a spectrum of phylogenetic and chemotaxonomic properties consistent with those of the members of the genus *Streptomyces*. The cell wall peptidoglycan was determined to contain LL-diaminopimelic acid. The predominant menaquinones were identified as MK-9(H_6_) and MK-9(H_8_), while the identified polar lipids consisted of aminolipid, diphosphatidylglycerol, glycolipid, hydroxyphosphatidylethanolamine, phospholipid, phosphatidylinositol, phosphatidylethanolamine, phosphatidylglycerol and lipid. The cell wall sugars consist of galactose, glucose and ribose. The predominant cellular fatty acids (>10.0%) were identified as iso-C_15:__0_ (34.8%) and anteiso-C_15:__0_(14.0%). Phylogenetic analysis identified that closely related strains for MUSC 164^T^ as *Streptomyces javensis* NBRC 100777^T^ (99.6% sequence similarity), *Streptomyces yogyakartensis* NBRC 100779^T^ (99.6%) and *Streptomyces violaceusniger* NBRC 13459^T^ (99.6%). The DNA–DNA relatedness values between MUSC 164^T^ and closely related type strains ranged from 23.8 ± 0.3% to 53.1 ± 4.3%. BOX-PCR fingerprints comparison showed that MUSC 164^T^ exhibits a unique DNA profile, with DNA G + C content determined to be 71.6 mol%. Based on the polyphasic study of MUSC 164^T^, it is concluded that this strain represents a novel species, for which the name *Streptomyces antioxidans* sp. nov. is proposed. The type strain is MUSC 164^T^ (=DSM 101523^T^ = MCCC 1K01590^T^). The extract of MUSC 164^T^ showed potent antioxidative and neuroprotective activities against hydrogen peroxide. The chemical analysis of the extract revealed that the strain produces pyrazines and phenolic-related compounds that could explain for the observed bioactivities.

## Introduction

Many therapeutic agents, such as antibiotics, anti-inflammatory and antioxidant compounds have been isolated from microorganisms (Bérdy, 2005; Williams, 2009). Discovery of these bacterial-derived bioactive compounds has a major impact on human health, helping people to live longer and reducing the mortality rate due to infectious and/or chronic diseases. In recent years, the accumulation of free radicals or oxidative stress has been identified as one of the major contribution to neuronal loss and occur early in all major neurodegenerative diseases (Lin and Beal, 2006; Fischer and Maier, 2015; Leszek et al., 2016). By reducing the presence of free radicals, increased intake of antioxidants is known to prevent and decrease the risk of these chronic diseases (Devasagayam et al., 2004; Bonda et al., 2010). Thus, continuous efforts have been directed toward searching for potent, natural antioxidants to prevent the deleterious effects of free radicals.

Over the years, exploring new taxa remains as one of the successful strategies which lead to discovery of therapeutic agents (Williams, 2009). As the most prolific producer of bioactive compounds, *Streptomyces* genus was initially proposed by Waksman and Henrici (1943) and metabolites isolated from these organisms have been shown to possess pharmaceutically relevant activities such as anti-inflammatory, antimicrobial, antioxidant activities (Bérdy, 2005; Wang et al., 2013; Kumar et al., 2014; Ser et al., 2015a, 2016a; Tan et al., 2016). Moreover, the metabolites derived from *Streptomyces* are described as potent protective agents in neuronal cells against oxidative stress induced damage. In fact, a recent study by Leiros et al. (2013) has identified seven bioactive compounds produced by *Streptomyces* sp. which protects against hydrogen peroxide (H_2_O_2_) challenge in primary cortical neurons. Unfortunately, many previous drug screening program focused on novel actinomycetes from terrestrial source, which in turn resulted in inefficient rediscovery of known bioactive compounds. Thus, researchers began to divert their attention to new or underexplored habitats, in hope to find new species that may yield promising bioactive compounds.

As one of the world's most dynamic environments, the mangrove ecosystem yields commercial forest products, supports coastal fisheries and protects coastlines (Alongi, 2008). Recently, there has been a renewed interest in the mangrove microorganisms' resources, considering that the changes in salinity and tidal gradient in the mangrove can trigger metabolic adaptations that could result in valuable metabolites production (Hong et al., 2009; Lee et al., 2014a; Azman et al., 2015). Several studies have discovered novel actinobacteria from the poorly explored mangrove environments, demonstrated by the isolation of *Streptomyces xiamenensis* (Xu et al., 2009), *Streptomyces sanyensis* (Sui et al., 2011), *Streptomyces qinglanensis* (Hu et al., 2012), *Streptomyces pluripotens* (Lee et al., 2014b), *Streptomyces gilvigriseus* (Ser et al., 2015b), and *Streptomyces mangrovisoli* (Ser et al., 2015a). Some of these novel strains are known to be bioactive strains as they were found to produce potent compounds with antibacterial, antifibrotic and antioxidant activities. Overall, these findings emphasized that these mangrove-derived Gram-positive filamentous bacteria could be potentially useful for discovery of new drugs or drug leads for neurodegenerative diseases which role of oxidative stress has been implicated, including Parkinson's diseases, Alzheimer's disease and multiple sclerosis.

In this study, a novel strain, MUSC 164^T^ was discovered from a mangrove soil located in east coast of Peninsular Malaysia. A polyphasic approach determined that MUSC 164^T^ represents a novel species of the *Streptomyces* genus, for which the name *Streptomyces antioxidans* sp. nov. is proposed. As a means to explore the bioactivities possessed by the strain, the extract of MUSC 164^T^ was subjected to several antioxidant assays prior to *in vitro* neuroprotective screening against hydrogen peroxide (H_2_O_2_). Gas chromatography-mass spectrometry (GC-MS) was used to perform chemical analysis for MUSC 164^T^ extract in order to reveal the chemical constituents present in the extract. Taken altogether, this study has implicated the potential of the mangrove-derived strain *Streptomyces antioxidans* sp. nov. in producing bioactive compounds, specifically with antioxidative and neuroprotective activities.

## Materials and methods

### Isolation and maintenance of isolate

Strain MUSC 164^T^ was recovered from a soil sample collected at site MUSC-TLS4 (3° 48′ 21.3″ N 103° 20′ 3.3″ E), located in the mangrove forest of Tanjung Lumpur in the state of Pahang, Peninsular Malaysia in December 2012. Topsoil samples of the upper 20 cm layer (after removing the top 2–3 cm) were collected and sampled into sterile plastic bags using an aseptic metal trowel, and stored at −20°C. Air-dried soil samples were ground with a mortar and pestle. Selective pretreatment of soil samples was performed using wet heat in sterilized water (15 min at 50°C; Takahashi et al., 1996). Five grams of the pretreated air-dried soil was mixed with 45 mL sterilized water and mill ground, spread onto the isolation medium ISP 2 (Shirling and Gottlieb, 1966) supplemented with cycloheximide (25 μg/mL) and nystatin (10 μg/mL), and incubated at 28°C for 14 days. Pure cultures of strain MUSC 164^T^ were isolated and maintained on slants of ISP 2 agar and in glycerol suspensions (20% v/v).

### Genomic and phylogenetic analyses

**Figure 1:**
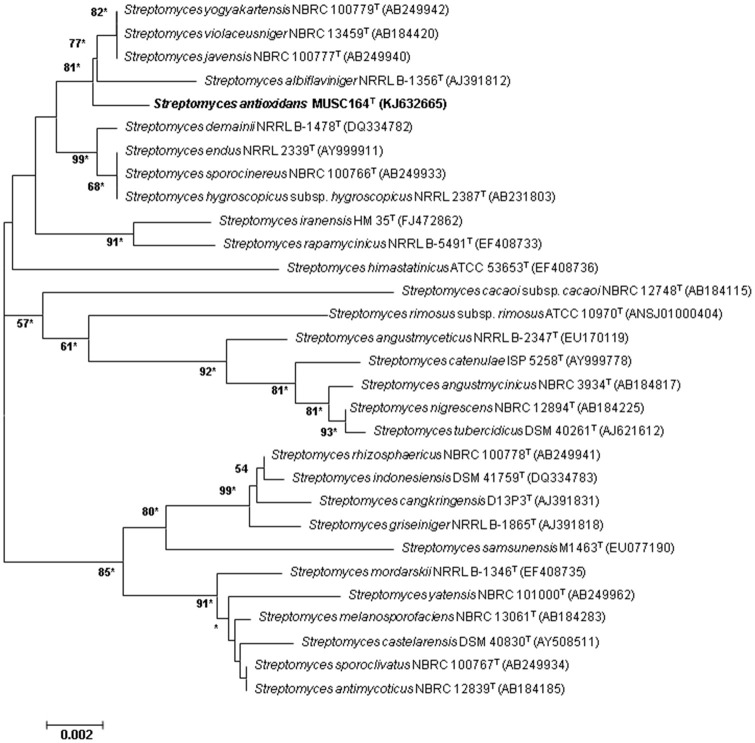
**Neighbor-joining phylogenetic tree based on almost complete 16S rRNA sequences (1491 nucleotides) showing the relationship between strain MUSC 164^**T**^ and representatives of some other related taxa**. Numbers at nodes indicate percentages of 1000 bootstrap re-samplings, only values above 50% are shown. Bar, 0.002 substitutions per site. Asterisks indicate that the corresponding nodes were also recovered using the maximum-likelihood tree-making algorithm.

Extraction of DNA was performed as previously described (Hong et al., 2009), followed by 16S rRNA gene amplification carried out as stated by Lee et al. (2014b). Using CLUSTAL-X software, the 16S rRNA gene sequence of strain MUSC 164^T^ was aligned with representative sequences of related type strains of the genus *Streptomyces* retrieved from the GenBank/EMBL/DDBJ databases (Thompson et al., 1997). Subsequently, the alignment was verified manually and adjusted before constructing the phylogenetic trees with the neighbor-joining (Saitou and Nei, 1987; Figure 1) and maximum-likelihood algorithms (Felsenstein, 1981; Figure S1), using the MEGA version 6.0 (Tamura et al., 2013). Evolutionary distances for the neighbor-joining algorithm were computed using Kimura's two-parameter model (Kimura, 1980). Calculations of sequence similarity was performed using EzTaxon-e server (http://eztaxon-e.ezbiocloud.net/) (Kim et al., 2012). The stability of the resultant trees topologies were evaluated by using the bootstrap resampling method of Felsenstein (1985).

BOX-PCR fingerprint analysis was carried out to characterize strain MUSC 164^T^ and the closely related strains using the primer BOX-A1R (5′-CTACGGCAAGGCGACGCTGACG-3′; Versalovic et al., 1991; Lee et al., 2014c). The PCR condition for BOX-PCR was performed as described by Lee et al. (2014d) and the PCR products were visualized by 2% agarose gel electrophoresis.

Genomic DNA extractions for DNA-DNA hybridization of strain MUSC 164^T^, *Streptomyces javensis* NBRC 100777^T^, *Streptomyces violaceusniger* NBRC 100779^T^ and *Streptomyces yogyakartensis* NBRC 13459^T^ were performed by the Identification Service of the DSMZ, Braunschweig, Germany following the protocol of Cashion et al. (1977). DNA-DNA hybridization was conducted as described by De Ley et al. (1970) with slight modifications described by Huss et al. (1983). The G + C content of strain MUSC 164^T^ was determined by HPLC (Mesbah et al., 1989).

### Chemotaxonomic characteristics

The analyses of peptidoglycan amino acid composition and sugars of strain MUSC 164^T^ were conducted by the Identification Service of the DSMZ using published protocols (Schumann, 2011). Analysis of respiratory quinones, polar lipids (Kates, 1986) and fatty acids (Sasser, 1990) were carried out by the Identification Service of the DSMZ. Major diagnostic cell wall sugars of strain MUSC 164^T^ were obtained as described by Whiton et al. (1985) and analyzed by TLC on cellulose plates (Staneck and Roberts, 1974).

### Phenotypic characteristics

**Figure 2:**
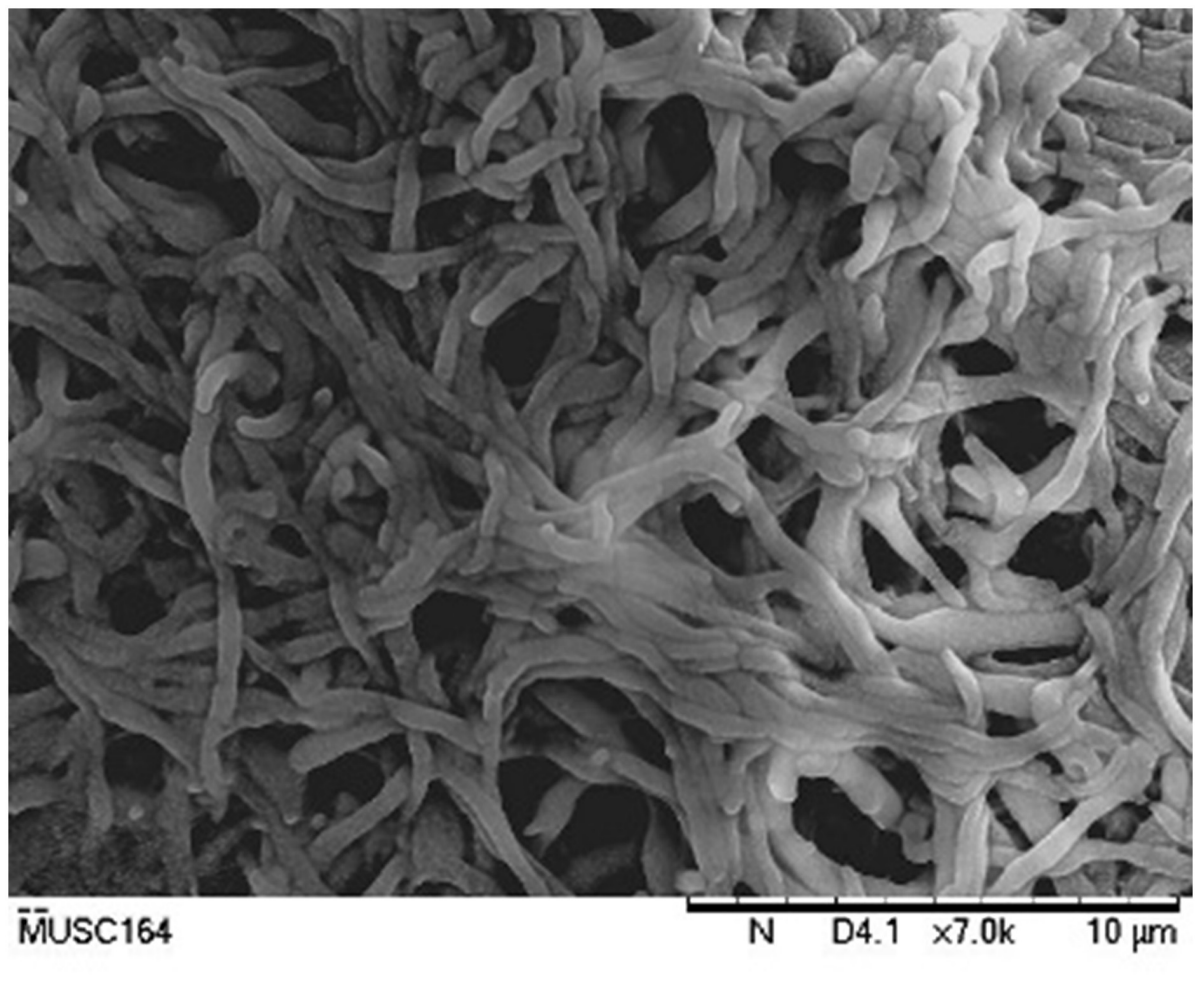
**Scanning electron microscope of ***Streptomyces antioxidans*** MUSC 164^**T**^**.

The cultural characteristics of strain MUSC 164^T^ were determined following growth on ISP 2, ISP 3, ISP 4, ISP 5, ISP 6, and ISP 7 agar (Shirling and Gottlieb, 1966), actinomycetes isolation agar (AIA; Atlas, 1993), *Streptomyces* agar (SA; Atlas, 1993), starch casein agar (SCA; Küster and Williams, 1964) and nutrient agar (Macfaddin, 2000) for 14 days at 28°C. The colony color was examined by using the ISCC-NBS color charts (Kelly, 1964). Light microscopy (80i, Nikon) and scanning electron microscopy (JEOL-JSM 6400) were utilized to evaluate the morphology of the strain after incubation on ISP 2 medium at 28°C for 7–14 days (Figure 2). Gram staining was performed and confirmed by using KOH lysis (Cerny, 1978). The growth temperature range was tested at 12–48°C (at intervals of 4°C) on ISP 2 agar, while the pH range for growth was tested in tryptic soy broth (TSB) between pH 4.0–10.0 (at intervals of 1.0 pH unit). Tolerance of NaCl was tested in TSB with concentrations ranging from 0 to 18% (w/v) at intervals of 2%. The responses to temperature, pH and NaCl were observed for 14 days. Catalase activity and production of melanoid pigments were determined following protocols described by Lee et al. (2014e). Hemolytic activity was examined after incubation at 32°C for 7–14 days using blood agar medium containing 5% (w/v) peptone, 3% (w/v) yeast extract, 5% (w/v) NaCl, and 5% (v/v) horse blood (Carrillo et al., 1996). Amylolytic, cellulase, chitinase, lipase, protease, and xylanase activities were determined by growing cells on ISP 2 medium as described by Meena et al. (2013). The carbon-source utilization and chemical sensitivity assays were determined using Biolog GenIII MicroPlates (Biolog, USA) according to the manufacturer's instructions.

All of the phenotypic assays mentioned were performed concurrently for strain MUSC 164^T^, *Streptomyces javensis* NBRC 100777^T^, *Streptomyces violaceusniger* NBRC 100779^T^ and *Streptomyces yogyakartensis* NBRC 13459^T^.

### Extract preparation of MUSC 164^T^

Seed medium was prepared by cultivating strain MUSC 164^T^ in TSB for 14 days prior to fermentation process. Fermentation was conducted in 500 mL Erlenmeyer flask containing 200 mL of sterile FM 3 medium, shaking at 200 rpm for 7–10 days at 28°C (Hong et al., 2009; Lee et al., 2012). The cell mass was separated by centrifugation at 12000 × *g* for 15 min and the supernatant was filtered and freeze-dried. The freeze-dried sample was extracted repeatedly with methanol. Subsequently, the extracting solvent was removed and concentrated by rotary vacuum evaporator at 40°C. The extract of MUSC 164^T^ was retrieved and suspended in dimethyl sulphoxide (DMSO) as vehicle reagent prior to bioactivity screening assays.

### Determination of antioxidant activity of MUSC 164^T^ extract

2,2-diphenyl-1-picrylhydrazyl (DPPH) scavenging activity by MUSC 164^T^ extract was determined using previous protocol with minor modification (Ser et al., 2015a). The reduction in radical is measured as decrease in the absorbance of 515 nm. Volume of 195 μL of 0.016% DPPH ethanolic solution was added to 5 μL of extract solution to make up final volume of 200 μL. Gallic acid was included as positive control. Reactions were carried out at room temperature in dark for 20 min before measurement with spectrophotometer at 515 nm. DPPH scavenging activity was calculated as follows:

$$\begin{array}{l} \text{DPPH scavenging activity }=\frac{\begin{array}{c} \text{Absorbance of control}\\ -\text{Absorbance of sample} \end{array}}{\text{Absorbance of control}}\times 100\% \end{array}$$

SOD activity was determined using SOD assay Kit − WST (Sigma-Aldrich) following manufacturer's instructions (Tan et al., 2015). Twenty microliter of sample solution was added to sample and blank 2 wells, while 20 μL of ddH_2_O was added to blank 1 and blank 3 wells. Subsequently, WST working solution (20 μL) was then added to each well followed by 20 μL of enzyme working solution to the sample and blank 1 wells. The resultant mixtures were mixed thoroughly and incubated at 37°C for 20 min. The absorbance was read at 450 nm and superoxide anion scavenging activity was calculated as follows:

$$\begin{array}{l} \text{SOD activity }=\\ \frac{\begin{array}{l} \left((\text{Absorbance of blank 1}-\text{Absorbance of blank 3}\right)\\ -(\text{Absorbance of sample}-\text{Absorbance of blank 2})) \end{array}}{\left(\text{Absorbance of blank 1}-\text{Absorbance of blank 3}\right)}\times \text{100}\% \end{array}$$

2,2′-azino-bis(3-ethylbenzothiazoline-6-sulphonic acid) (ABTS) assay was performed as previously described in published literature with some modifications (Miser-Salihoglu et al., 2013; Ser et al., 2016b). ABTS radical cation (ABTS•) was generated by reacting ABTS stock solution (7 mM) and potassium persulphate (2.45 mM) for 24 h prior to assay. The change in radical amount was indicated by decrease in absorbance at 743 nm. Metal-chelating activity was measured as described by Manivasagan et al. (2013) with slight modification. 2 mM of FeSO_4_was added to extract and the reaction was initiated by adding 5 mM of ferrozine before measuring at 562 nm using spectrophotometer.

### Cell lines maintenance and growth condition

Neuronal SH-SY5Y cells were maintained in DMEM media (supplemented with 10% FBS) in humidified incubator (5% CO2 in air at 37°C) as described by Wong et al. (2012) with minor modification.

### Assessment of toxicity and neuroprotective activity of MUSC 164^T^ extract

Confluence cells were harvested and seeded at a density of 3 × 10^4^ cells/well into a sterile flat bottom 96-well plate. The seeded cells were allowed to adhere for 48 h before the experiment. Firstly, toxicity of the extract was investigated by treating the cells MUSC 164^T^ extract (0–400 μg/mL) for 24 h prior to measurement of cell viability using MTT assay (Chan et al., 2012). On the other hand, for evaluation of neuroprotective activity, cells were pretreated with MUSC 164^T^ (0–400 μg/mL) extract for 2 h prior to treatment with H_2_O_2_ (Wong et al., 2012). For these screenings, media with DMSO was included as negative control along with a non-inoculated extract. Subsequently, MTT assay was performed 24 h after H_2_O_2_ treatment. Tetrazolium salt solution was added into each well and incubated further for 4 h. Plates were analyzed in microplate reader at 570 nm (with a reference wavelength of 650 nm). The percentage of cell viability was calculated as follows:

$$\begin{array}{l} \text{Percentage of cell viability }=\frac{\text{Absorbance of treated cells}}{\text{Absorbance of untreated cells}}\times 100\text{\%} \end{array}$$

### Gas chromatography-mass spectrometry (GC-MS) analysis

GC-MS analysis was performed in accordance with our previous developed method with slight modification (Supriady et al., 2015; Ser et al., 2015a). The machine used was Agilent Technologies 6980N (GC) equipped with 5979 Mass Selective Detector (MS), HP-5MS (5% phenyl methyl siloxane) capillary column of dimensions 30.0 m × 250 × 0.25 μm and used helium as carrier gas at 1 mL/min. The column temperature was programmed initially at 40°C for 10 min, followed by an increase of 3°C/min to 250°C and was kept isothermally for 5 min. The MS was operating at 70 eV. The constituents were identified by comparison of their mass spectral data with those from NIST 05 Spectral Library.

### Statistical analysis

Experiments to evaluate bioactivities were performed in quadruplicate and all results were expressed as mean ± standard deviation (SD). Statistical analysis was performed using one-way analysis of variance (ANOVA) with SPSS statistical analysis software. A difference was considered statistically significant when *p* ≤ 0.05.

## Results and discussion

Biologically active compounds isolated from microorganisms remain to be vital for development of new drugs, particularly pharmaceutical and agricultural industry (Bérdy, 2005; Jensen et al., 2005). Oxidative stress has been implicated in pathogenesis of various chronic human disease, particularly neurodegenerative diseases (Radi et al., 2014). An imbalance in free radicals production and antioxidant mechanisms causes modifications and damages on biological macromolecules including protein, lipid and DNA. These detrimental effects of free radicals eventually lead to neuronal cell loss–a scenario which is commonly seen in neurodegenerative diseases (Uttara et al., 2009). Antioxidants are responsible of removing or reducing amount of these harmful radicals; high intake of antioxidants have been associated with reduced risk of developing neurodegenerative diseases (Bonda et al., 2010; Lassmann and van Horssen, 2015). The need to search for novel, potent antioxidative agents to combat against these diseases has called upon researchers to venture into new or underexplored habitats for the discovery of bioactive strains that could produce potent antioxidant(s) (Harvey, 2000; Penesyan et al., 2010). In current study, the mangrove forest soil-derived MUSC 164^T^ strain shows abundant growth on ISP 2, ISP 3, ISP 5, ISP 6, ISP 7 agar, actinomycetes isolation agar, nutrient agar and starch casein agar after 7–14 days at 28°C. The strain grows moderately on *Streptomyces* agar, and does not grow on ISP 4 agar. The colors of the aerial and substrate mycelium were media-dependent as indicated by Table S1. Both aerial and vegetative hyphae were abundant, well developed and not fragmented as observed observed from 14-day-old culture grown on ISP 2 agar. These morphological features are consistent with assignment of the strain to the genus *Streptomyces* (Williams et al., 1989). Growth was found to occur at 26–36°C (optimum 28–32°C), with 0–6% NaCl tolerance (optimum 0–2%) and at pH 6.0–8.0 (optimum pH 7.0). Cells were found to be positive for catalase but lack of hemolytic activity and melanoid pigment production. Furthermore, cells were capable of hydrolyzing soluble starch and carboxymethylcellulose, but unable to hydrolyze casein, chitin, tributyrin (lipase) and xylan. Using a range of phenotypic properties, strain MUSC 164^T^ can be differentiated from closely related members of the genus *Streptomyces* (Table 1). Furthermore, chemical sensitivity assays showed that the strain was resistant to aztreonam, fusidic acid, guanine HCl, lincomycin, lithium chloride, minocycline, nalidixic acid, niaproof 4, potassium tellurite, rifamycin RV, sodium bromate, sodium butyrate, 1% sodium lactate, tetrazolium blue, tetrazolium violet, troleandomycin, and vancomycin.

**Table 1:** **Differentiation characteristics of strain MUSC 164^**T**^ and type strains of phylogenetically closely related species of the genus ***Streptomyces*****.

**Characteristic**	**1**	**2**	**3**	**4**
**MORPHOLOGY (ON ISP 2):**				
Color of aerial mycelium	Yellowish white	Yellowish white	Pale orange yellow	Vivid greenish yellow
Color of substrate mycelium	Brilliant greenish yellow	Light yellow	Brilliant yellow	Brilliant Greenish yellow
**GROWTH AT:**				
26°C	+	+	+	(+)
36°C	(+)	+	+	−
pH 8	(+)	−	−	+
4% NaCl	(+)	+	+	−
Catalase	+	+	+	+
Hemolytic	−	−	−	−
**HYDROLYSIS OF:**				
Casein (protease)	−	+	−	+
Tributyrin (lipase)	−	+	+	+
Starch (amylolytic)	+	+	+	+
Carboxymethylcellulose (cellulase)	+	+	+	+
Xylan (xylanase)	−	+	+	+
**CARBON SOURCE UTILIZATION:**				
Dextrin	−	+	+	+
Gentiobiose	−	−	+	+
Sucrose	+	−	−	−
D−turanose	+	−	−	−
Stachyose	+	−	−	−
α−D−lactose	−	+	+	+
D−melibiose	−	−	+	+
N−acetyl−neuraminic acid	+	−	−	−
D−mannose	−	−	+	+
D−fructose	−	−	+	+
D−galactose	−	−	+	−
L−fucose	−	+	+	+
Inosine	−	−	+	+
D−mannitol	−	+	+	+
p−hydroxy−phenylacetic acid	+	−	−	−
acetoacetic acid	+	−	−	−
**CHEMICAL SENSITIVITY ASSAYS:**				
Troleandomycin	+	−	−	−
Vancomycin	+	−	−	−
Fusidic acid	+	−	−	−
Lincomycin	+	−	−	−
Niaproof 4	+	−	−	−
Lithium chloride	+	−	−	−
Guanidine HCl	+	−	−	−

*Strains: 1, Streptomyces antioxidans sp. nov. MUSC 164^T^; 2, Streptomyces javensis NBRC 100777^T^; 3, Streptomyces yogyakartensis NBRC 100779^T^; 4, Streptomyces violaceusniger NBRC 13459^T^. All data were obtained concurrently in this study. +, Positive; −, negative; (+), weak*.

*All strains are positive for utilization of D-cellobiose, D-raffinose, N-acetyl-D-glucosamine, α-D-glucose, gelatin, glucuronamide, γ-amino-butyric acid, α-hydroxy-butyric acid, β-hydroxy-D,L-butyric acid, and α-keto-butyric acid. All strains are negative for assimilation of D-maltose, β -methyl-D-glucoside, D-salicin, N-acetyl- β -D-mannosamine, N-acetyl-D-galactosamine, 3-methyl glucose, D-fucose, D-sorbitol, D-glucose-6-PO4, D-serine and citric acid*.

The nearly complete 16S rRNA gene sequence was obtained for strain MUSC 164^T^ (1491 bp; GenBank/EMBL/DDBJ accession number KJ632665) and phylogenetic trees were reconstructed to determine the phylogenetic position of this strain (Figure 1, Figure S1). Phylogenetic analysis exhibited that closely related strains include *Streptomyces javensis* NBRC 100777^T^, *Streptomyces yogyakartensis* NBRC 100779^T^ and *Streptomyces violaceusniger* NBRC 13459^T^, as they formed a distinct clade at high bootstrap value of 81% (Figure 1). The analysis of 16S rRNA gene sequence for strain MUSC 164^T^ exhibited highest similarity to strain *Streptomyces javensis* NBRC 100777^T^ (99.6% sequence similarity), *Streptomyces yogyakartensis* NBRC 100779^T^ (99.6%) and *Streptomyces violaceusniger* NBRC 13459^T^ (99.6%); while the type strains of other species of the genus *Streptomyces* showed sequences similarities below 99.3%. The DNA–DNA relatedness values between strain MUSC 164^T^ and *Streptomyces javensis* NBRC 100777^T^ (23.8 ± 0.2%), *Streptomyces yogyakartensis* NBRC 100779^T^ (30.2 ± 2.7%) and *Streptomyces violaceusniger* NBRC 13459^T^ (53.1 ± 4.4%) were significantly below 70% which was reported as the threshold value for the delineation of bacterial species (Wayne et al., 1987). The BOX-PCR analysis revealed a unique fingerprint pattern by strain MUSC 164^T^ as compared with its closely related type strains (Figure S2). These results further supported the results of DNA-DNA hybridizations and phylogenetic analysis, which indicated the novel status of strain MUSC 164^T^ in the genus *Streptomyces*.

The major cellular fatty acids in MUSC 164^T^ were identified as iso-C_15:__0_ (34.8%) and anteiso-C_15:__0_ (14.0%) (Table 2). The fatty acids profile of MUSC 164^T^ displayed some levels of similarities with those of closely related phylogenetic neighbors such as *Streptomyces javensis* NBRC 100777^T^, *Streptomyces yogyakartensis* NBRC 100779^T^ and *Streptomyces violaceusniger* NBRC 13459^T^, as they contain iso-C_15:__0_ (24.1–36.3%) as their predominant fatty acids. Nonetheless, the fatty acid profile of MUSC 164^T^ was quantitatively different from those of these type strains; for instance, iso-C_15:__0_(34.8%) was found to be predominant in strain MUSC 164^T^ (Table 2), but the amount of the same fatty acid was much lesser in *Streptomyces yogyakartensis* NBRC 100779^T^ (24.1%). The polar lipids of MUSC 164^T^ were aminolipid, diphosphatidylglycerol, glycolipid, hydroxyphosphatidylethanolamine, phospholipid, phosphatidylinositol, phosphatidylethanolamine, phosphatidylglycerol and lipid. The differences in polar lipid profiles indicated that MUSC 164^T^ differs from related type strains; for example, strain MUSC 164^T^ contain aminolipid and glycolipid (Figure S3A) that were not detected in *Streptomyces javensis* NBRC 100777^T^ (Figure S3B).

**Table 2:** **Cellular fatty acid composition of strain MUSC 164^**T**^ and its closely related ***Streptomyces*** species**.

**Fatty acid**	**1**	**2**	**3**	**4**
iso-C_11:0_	−	0.1	−	−
C_12:0_	0.1	0.1	−	−
iso-C_13:0_	1.5	1.0	0.5	0.5
anteiso-C_13:0_	0.2	0.1	−	0.1
C_13:0_	0.1	−	−	−
iso-C_14:0_	2.5	1.7	3.1	3.8
C_14:0_	0.6	0.4	0.3	0.3
iso-C_15:0_	34.8	36.3	24.1	32.5
anteiso-C_15:0_	14.0	10.1	5.5	6.9
C_15:1_B	1.2	0.1	0.6	0.2
C_15:0_	3.7	0.7	1.4	0.7
iso-C_16:1_ H	0.9	1.1	3.5	2.2
iso-C_16:0_	7.0	9.1	21.3	15.1
C_16:1_ Cis 9	6.4	3.7	4.3	3.8
anteiso-C_15:0_ 2OH	0.3	−	−	−
C_16:0_	5.9	5.4	4.4	4.4
iso-C_15:0_ 3OH	0.1	−	−	−
C_16:0_ 9Methyl	5.3	9.1	10.4	10.0
anteiso-C_17:1_C	1.1	1.2	1.2	1.1
iso-C_17:0_	7.6	13.9	10.1	13.8
anteiso-C_17:0_	3.9	4.8	3.1	3.3
C_17:1_Cis 9	1.1	0.3	1.4	0.2
C_17:0_Cyclo	0.7	0.2	1.1	0.4
C_17:0_	0.8	0.3	0.5	0.1
C_17:0_10Methyl	−	−	1.0	0.1
iso-C_18:1_H	−	0.1	1.3	0.2
iso-C_18:0_	−	0.1	0.3	0.2
iso-C_17:0_ 2OH	−	−	0.1	−
C_18:0_	−	0.1	0.1	0.2
iso-C_19:0_	−	−	−	0.1

*Strains: 1, Streptomyces antioxidans sp. nov. MUSC 164^T^; 2, Streptomyces javensis NBRC 100777^T^; 3, Streptomyces yogyakartensis NBRC 100779^T^; 4, Streptomyces violaceusniger NBRC 13459^T^. −, < 0.1% or not detected. All data are obtained concurrently from this study*.

Strain MUSC 164^T^ presented a type I cell-wall as it contains LL-diaminopimelic acid (Lechevalier and Lechevalier, 1970), an amino acid which has been found in many species of the genus *Streptomyces* (Lee et al., 2005, 2014b; Xu et al., 2009; Hu et al., 2012; Ser et al., 2015a, Ser et al., 2015b). The predominant menaquinones of strain MUSC 164^T^ were detected as MK-9(H_6_) (51%) and MK-9(H_8_) (39%). These findings parallel those reported by Kim et al. (2003). The cell wall sugars detected were galactose, glucose and ribose. The G + C content of strain MUSC 164^T^ was 71.6 mol% which falls within the range of 67.0–78.0 mol% described for species of the genus *Streptomyces* (Kim et al., 2003).

Based on the results of phylogenetic analysis, DNA-DNA hybridization, chemotaxonomic and phenotypic analysis, it is evident that strain MUSC 164^T^ is different from all other species in the genus *Streptomyces*; the strain represents a novel species within the genus *Streptomyces*, for which the name *Streptomyces antioxidans* sp. nov. is proposed. As an attempt to explore bioactivities possessed by the strain, MUSC 164^T^ extract was subjected to several antioxidant assays. DPPH assay revealed the ability of MUSC 164^T^ to produce free radical scavenging compound(s) as 2 mg/mL of extract showed significant activity at 18.31 ± 2.03% (Table 3). Similarly, the extract was able to reduce ABTS radical and chelate ferrous ion significantly with highest activity recorded to be 30.38 ± 2.27 and 43.66 ± 0.98%, respectively. Additionally, SOD assay suggested antioxidant(s) in MUSC 164^T^ extract with ranging antioxidant activity of 53.09–79.84%, depending on extract concentration. In summary, all the antioxidant assays suggested the potential of MUSC 164^T^ to produce potent antioxidant(s) that could scavenge free radicals and may reduce occurrence of oxidative stress.

**Table 3:** **Radical scavenging activity of MUSC 164^**T**^ evaluated using different antioxidant assays**.

**Antioxidant assays**	**Concentration of MUSC 164^T^ extract (mg/mL)**	**Mean ± standard deviation (%)**
DPPH	0.125	ND
	0.25	ND
	0.5	2.42 ± 1.58^*^
	1.0	6.66 ± 1.91^*^
	2.0	18.31 ± 2.03^*^
ABTS	0.125	5.14 ± 0.94^*^
	0.25	8.27 ± 1.63^*^
	0.5	10.63 ± 0.54^*^
	1.0	18.14 ± 1.72^*^
	2.0	30.38 ± 2.27^*^
Superoxide dismutase-like	0.09375	53.09 ± 2.53^*^
	0.1875	56.92 ± 1.71^*^
	0.375	69.46 ± 0.64^*^
	0.75	69.94 ± 1.51^*^
	1.5	79.84 ± 1.16^*^
Metal-chelating	0.125	NA
	0.25	14.41 ± 2.08^*^
	0.5	20.48 ± 1.58^*^
	1.0	29.29 ± 0.66^*^
	2.0	43.66 ± 0.98^*^

Symbol (^*^) indicates p < 0.05 significant difference between MUSC 164^T^ extract and controls (without MUSC 164^T^ extract).

* *NA, not available, ND, not detected*.

**Figure 3:**
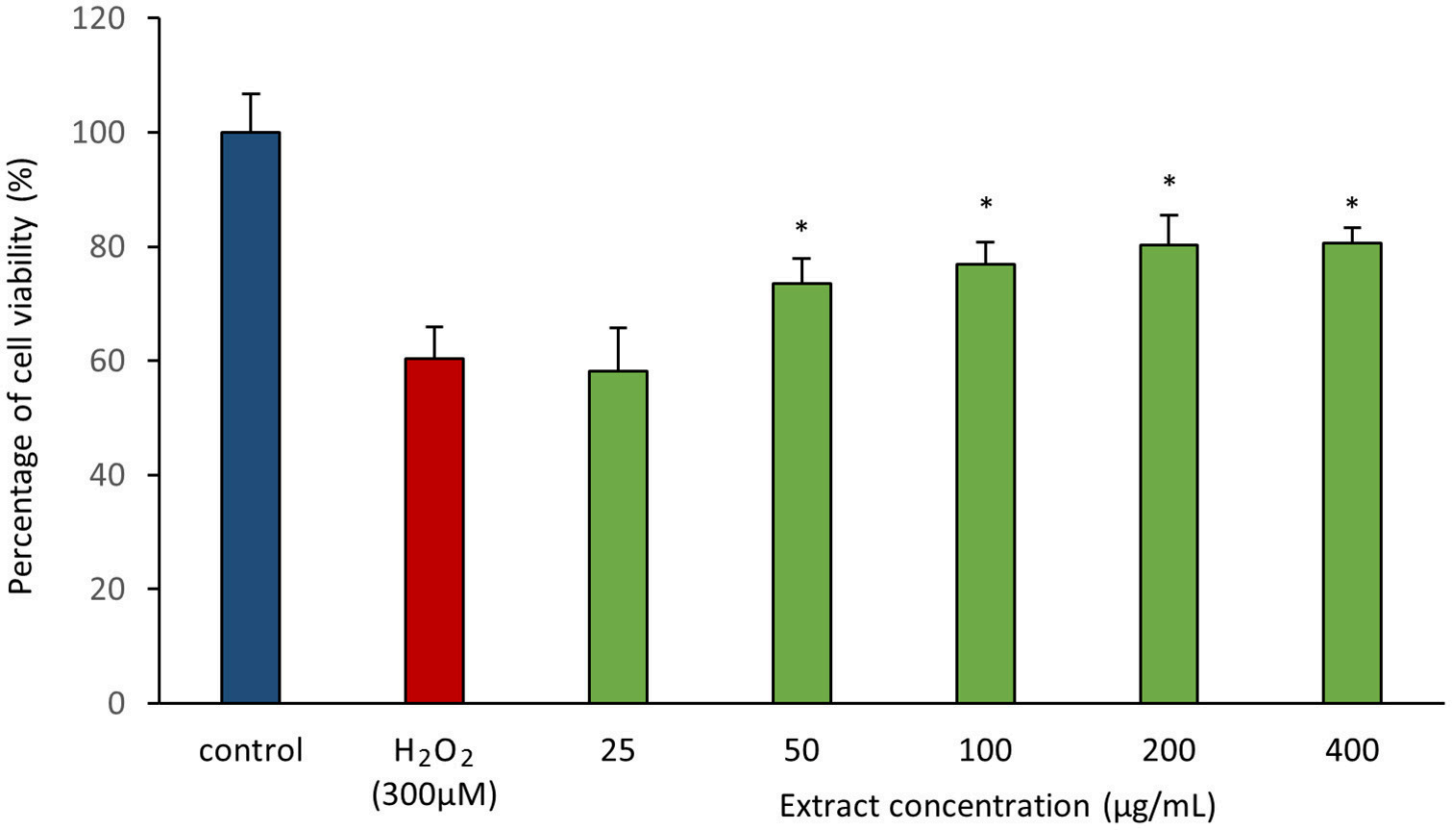
**Neuroprotective activity of MUSC 164^**T**^ extract against hydrogen peroxide (H_**2**_O_**2**_) in SH-SY5Y cells**. The measurement of cell viability was done using MTT assay. Media containing 0.5% DMSO was used as control. All data are expressed as mean ± standard deviation. Symbol (^*^) indicates *p* < 0.05 significant difference between the inducer and cells treated with MUSC 164^T^ extract.

As antioxidant assays revealed presence of antioxidant(s) in MUSC 164^T^ extract, *in vitro* cellular screening assay was then performed. The aim of this screening is to investigate if the extract is capable of protecting neuronal cells against the oxidative stress cellular damage elicited by an oxidative stress inducer, H_2_O_2_. Even though with short half-life, H_2_O_2_ is highly soluble in water and dissociates to hydroxyl and superoxide ions, which leads to oxidative damage to important macromolecules and ultimately cell death (Triana-Vidal and Carvajal-Varona, 2013). Thus, this molecule has been used widely in oxidative studies (Whittemore et al., 1995; Suematsu et al., 2011; Wong et al., 2012; Triana-Vidal and Carvajal-Varona, 2013). Initially, the viability of SH-SY5Y cells was examined following exposure to the extract up to 400 μg/mL and no significant toxic effect was observed in SH-SY5Y (i.e., cell viability remains >98%). In subsequent neuroprotective experiment, the obtained results have demonstrated MUSC 164^T^ extract confers a significant level of protection on neuronal cells when challenged with H_2_O_2_. Highest cell viability was recorded at 80.62 ± 2.75% when neuronal cells were pre-treated with 400 μg/mL of extract (Figure 3) prior to induction by H_2_O_2_. As antioxidant assays suggested presence of antioxidant(s) in the extract, these compounds can possibly quench H_2_O_2_, preventing oxidative damage against the neuronal cells. Previous study has showed that compounds produced by *Streptomyces* sp. protect primary cortical neurons against oxidative stress, accompanied by alteration in the expression of apoptotic genes and transcription factor for antioxidant pathways (Leiros et al., 2013). Thus, further study on underlying neuroprotective mechanisms induced by MUSC 164^T^ would be helpful for the development of new drugs in preventing the occurrence of neurodegenerative disorders.

**Figure 4:**
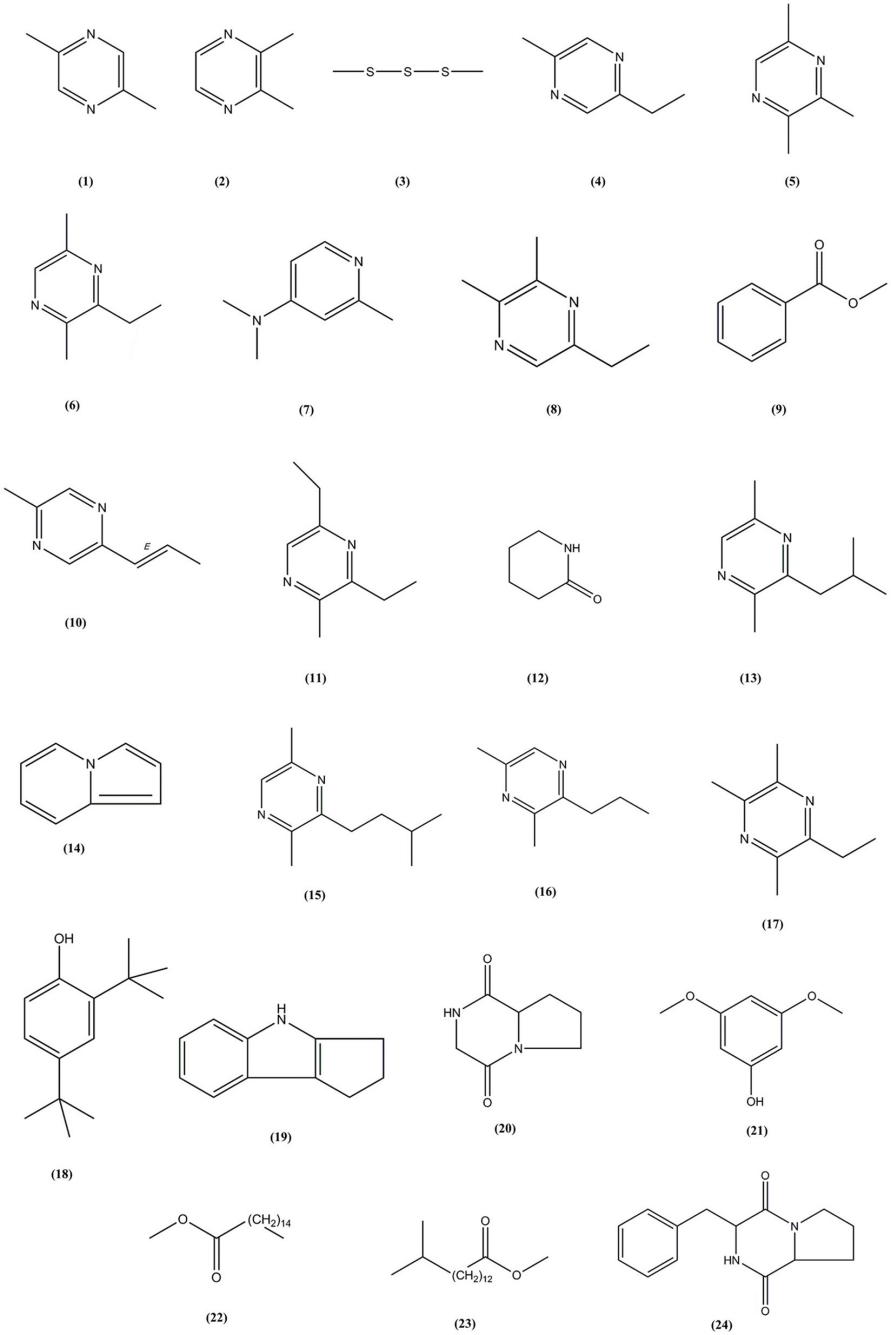
**Chemical structures of constituents detected in MUSC 164^**T**^ extract**.

Following the evaluation of bioactivities possessed by MUSC 164^T^ extract, GC-MS was performed to assist with the chemical profiling and to identify compounds present in the extract. This technique enables identification of compounds present in *Streptomyces* extract as GC separates the compounds and MS generates characteristic mass profile for each of the compounds present (Pollak and Berger, 1996; Karanja et al., 2010; Jog et al., 2014). Our results revealed 24 compounds present in MUSC 164^T^ extract (Table 4) and their chemical structures (Figure 4, Figure S4) as Pyrazine, 2,5-dimethyl- (**1**), Pyrazine, 2,3-dimethyl- (**2**), Dimethyl trisulfide (**3**), Pyrazine, 2-ethyl-5-methyl- (**4**), Pyrazine, trimethyl- (**5**), Pyrazine, 3-ethyl-2,5-dimethyl- (**6**), 4-Pyridinamine, N,N,2-trimethyl- (**7**), 2,3-Dimethyl-5-ethylpyrazine (**8**), Benzoic acid, methyl ester (**9**), Pyrazine, 2-methyl-5-(1-propenyl)-, (E)- (**10**), Pyrazine, 3,5-diethyl-2-methyl- (**11**), 2-Piperidinone (**12**), Pyrazine, 2,5-dimethyl-3-(2-methylpropyl)- (**13**), Indolizine (**14**), Pyrazine, 2,5-dimethyl-3-(3-methylbutyl)- (**15**), Pyrazine, 3,5-dimethyl-2-propyl- (**16**), 2,3,5-Trimethyl-6-ethylpyrazine (**17**), Phenol, 2,4-bis(1,1-dimethylethyl)- (**18**), 1,2,3,4-Tetrahydro-cyclopenta[b]indole (**19**), Pyrrolo[1,2-a]pyrazine-1,4-dione, hexahydro- (**20**), Phenol, 3,5-dimethoxy- (**21**), Hexadecanoic acid, methyl ester (**22**), Pentadecanoic acid, 14-methyl, methyl ester (**23**) and Pyrrolo[1,2-a]pyrazine-1,4-dione, hexahydro-3-(2-phenylmethyl)- (**24**).

**Table 4:** **Compounds identified from MUSC 164^**T**^ extract using GC-MS**.

**No**	**Retention time (min)**	**Compound**	**Formula**	**Molecular weight (MW)**	**Quality (%)**
1	13.484	Pyrazine, 2,5-dimethyl-	C_6_H_8_N_2_	108.14	90
2	14.022	Pyrazine, 2,3-dimethyl-	C_6_H_8_N_2_	108.14	80
3	17.111	Dimethyl trisulfide	C_2_H_6_S_3_	126.26	78
4	19.526	Pyrazine, 2-ethyl-5-methyl-	C_7_H_10_N_2_	122.17	90
5	19.595	Pyrazine, trimethyl-	C_7_H_10_N_2_	122.17	78
6	24.218	Pyrazine, 3-ethyl-2,5-dimethyl-	C_8_H_12_N_2_	136.19	94
7	24.544	4-Pyridinamine, N,N,2-trimethyl-	C_8_H_12_N_2_	136.19	53
8	24.630	2,3-Dimethyl-5-ethylpyrazine	C_8_H_12_N_2_	136.19	91
9	24.985	Benzoic acid, methyl ester	C_8_H_8_O_2_	136.15	90
10	25.265	Pyrazine, 2-methyl-5-(1-propenyl)-, (E)-	C_8_H_10_N_2_	134.18	59
11	28.464	Pyrazine, 3,5-diethyl-2-methyl-	C_9_H_14_N_2_	150.22	72
12	29.540	2-Piperidinone	C_5_H_9_NO	99.13	59
13	30.701	Pyrazine, 2,5-dimethyl-3-(2-methylpropyl)-	C_10_H_16_N_2_	164.25	80
14	34.970	Indolizine	C_8_H_7_N	117.15	64
15	36.057	Pyrazine, 2,5-dimethyl-3-(3-methylbutyl)-	C_11_H_18_N_2_	178.27	83
16	37.024	Pyrazine, 3,5-dimethyl-2-propyl-	C_9_H_14_N_2_	150.22	64
17	44.142	2,3,5-Trimethyl-6-ethylpyrazine	C_9_H_14_N_2_	150.22	68
18	44.474	Phenol, 2,4-bis(1,1-dimethylethyl)-	C_14_H_22_O	206.32	95
19	46.969	1,2,3,4-Tetrahydro-cyclopenta[b]indole	C_11_H_11_N	157.21	87
20	53.251	Pyrrolo[1,2-a]pyrazine-1,4-dione, hexahydro-	C_7_H_10_N_2_O_2_	154.17	97
21	56.158	Phenol, 3,5-dimethoxy-	C_8_H_10_O_3_	154.16	58
22	58.041	Hexadecanoic acid, methyl ester	C_17_H_34_O_2_	270.45	94
23	59.242	Pentadecanoic acid, 14-methyl, methyl ester	C_17_H_34_O_2_	270.45	90
24	72.065	Pyrrolo[1,2-a]pyrazine-1,4-dione, hexahydro-3-(2-phenylmethyl)-	C_14_H_16_N_2_O_2_	244.29	97

Through GC-MS, most of the compounds were identified to be heterocyclic organic compounds. Naturally occurring phenolic compounds have been widely accepted as potent antioxidants and these compounds are believed to play an important role in the prevention of chronic diseases resulting from oxidative stress (Soobrattee et al., 2005; Gülcin and Beydemir, 2013). The phenolic compound **18** has been detected in previously isolated *Streptomyces* sp. from vermicompost and mangrove forest (Narendhran et al., 2014; Tan et al., 2015; Ser et al., 2015a,c). This compound has been shown to exhibit antioxidant activity, probably owing to its hydrogen-donating ability (Brewer, 2011; Narendhran et al., 2014). On the other hand, pyrazines are heterocyclic compounds that contain two nitrogen atoms in their aromatic ring; some of these compounds are known to exhibit various bioactivities including antimicrobial, anticancer, antioxidant, neuroprotection against ischemia/reperfusion injuries and hypoxia (Premkumar and Govindarajan, 2005; Jia et al., 2009; Baldwin et al., 2013; Tan et al., 2015; Ser et al., 2015a,c). Previous studies have demonstrated that microorganisms are capable of producing compounds **1**, **2**, **5**, **6**, **15**, and **17** with antioxidant activity (Sun et al., 2013; Citron et al., 2015; Pongsetkul et al., 2015). *Bacillus methylotrophicus* KOSM11 was used in fermentation industry for traditional soybean paste and found to produce compound **1** and **5**; the presence of these pyrazines and their related compounds in various food and plants have been linked with antioxidant activities (Liu et al., 2012; Sun et al., 2013; Xu et al., 2015). Furthermore, two pyrrolopyrazines were observed in MUSC 164^T^ extract, which are compound **20** and **24**. These compounds were previously detected in several *Streptomyces* sp. and they have been associated with antioxidant activity exhibited by these strains (Gopi et al., 2014; Tan et al., 2015; Ser et al., 2015a,c). Gopi et al. (2014) has also reported that these compounds were highly capable of scavenging or reducing amount of free radicals when assessed with reducing power assay. Thus, the detection of these heterocyclic compounds present in MUSC 164^T^ could account for the antioxidant activity and protect SH-SY5Y cells against H_2_O_2_ insults.

The current study described a novel streptomycete designated as MUSC 164^T^ that produces a mixture of compounds, with some of them could be responsible for the free radical scavenging activities detected via several antioxidant assays. Furthermore, the extract has demonstrated its potential in conferring neuroprotection against oxidative insults, possibly by preventing oxidative stress and activating of antioxidant defense systems that are crucial for survival of SH-SY5Y cells. In conclusion, these preliminary studies have revealed the antioxidative and *in vitro* neuroprotective properties of MUSC 164^T^ which merit for further investigations focusing on the isolation and characterization of chemical compounds using bioassay-guided purification. The identified bioactive principles could essentially be important for the development of pharmacological agents for neurodegenerative diseases.

## Description of *Streptomyces antioxidans* sp. nov.

*Streptomyces antioxidans* sp. nov. (an.ti.o′xi.dans. Gr. pref. anti, against; N.L. v. oxidare, to oxidize; N.L. part. adj. antioxidans, non-oxidizing, referring to the antioxidant properties of this strain).

Gram-positive actinobacteria that forms yellowish-white aerial and brilliant greenish yellow substrate mycelium on ISP 2 agar. The colors of the aerial and substrate mycelium are media-dependent (Table S1).

Abundant growth was observed on ISP 2, ISP 3, ISP 5, ISP 6, ISP 7, actinomycetes isolation agar, starch casein agar and nutrient agar after 7–14 days at 28°C; cells grow moderately on *Streptomyces* agar, and does not grow on ISP 4 agar. Cells grow at 26–36°C (optimum 28–32°C), pH 6.0–8.0 (optimum pH 7.0), with 0–6% NaCl tolerance (optimum 0–2%). Cells are positive for catalase but negative for hemolytic activity and melanoid pigment production. Soluble starch and carboxymethylcellulose are hydrolyzed but casein, chitin, xylan, and tributyrin (lipase) are not. The following compounds are utilized as sole carbon sources: acetic acid, acetoacetic acid, α-D-glucose, α-hydroxy-butyric acid, α-keto-butyric acid, α-keto-glutaric acid, β-hydroxyl-D,L-butyric acid, bromo-succinic acid, D-cellobiose, D-fructose-6-phosphate, D-galactose, D-galacturonic acid, D-glucuronic acid, D-lactic acid methyl ester, D-malic acid, D-raffinose, D-saccharic acid, D-trehalose, D-turanose, formic acid, gelatin, glucuronamide, L-galactonic acid lactone, L-lactic acid, L-rhamnose, N-acetyl-D-glucosamine, N-acetyl-neuraminic acid, pectin, p-hydroxyl-phenylacetic acid, quinic acid, stachyose, sucrose, Tween 40, γ-amino-butyric acid, and myo-inositol. The following compounds are not utilized as sole carbon sources: α-D-lactose, β-methyl-D-glucoside, citric acid, D-arabitol, D-aspartic acid, Dextrin, D-fructose, D-fucose, D-glucose-6-phosphate, D-gluconic acid, D-maltose, D-mannitol, D-mannose, D-melibiose, D-salicin, D-serine, D-sorbitol, gentiobiose, glycerol, glycyl-L-proline, inosine, L-fucose, L-malic acid, methyl pyruvate, mucic acid, N-acetyl-β-D-mannosamine, N-acetyl-D-galactosamine, propionic acid, and 3-methyl glucose. The following compounds are not utilized as sole carbon sources: L-alanine, L-arginine, L-aspartic acid, L-histidine, L-pyroglutamic acid; while L-glutamic acid and L-serine are utilized as sole nitrogen sources.

The cell wall peptidoglycan contains LL-diaminopimelic acid. The predominant menaquinones are MK-9(H_6_) and MK-9(H_8_). The polar lipids consist of aminolipid, diphosphatidylglycerol, glycolipid, hydroxyphosphatidylet hanolamine, phospholipid, phosphatidylinositol, phosphatidylethanolamine, phosphatidylglycerol, and lipid. The cell wall sugars are galactose, glucose and ribose. The major cellular fatty acids are iso-C_15:__0_ and anteiso-C_15:__0_.

The type strain, MUSC 164^T^ (=DSM 101523^T^ = MCCC 1K01590^T^) was isolated from mangrove soil collected from the Tanjung Lumpur mangrove forest (state of Pahang, Peninsular Malaysia). The 16S rRNA gene sequence of strain MUSC 164^T^ has been deposited in GenBank/EMBL/DDBJ under the accession number KJ632665. The G + C content of the genomic DNA of the type strain is 71.6 mol%.

## Author contributions

The experiments, data analysis and manuscript writing were performed by H-LS, LT-HT, B-HG, and L-HL, UDP, SNAM, W-FY, and K-GC provided vital guidance and technical support. L-HL and B-HG founded the research project.

### Conflict of interest statement

The authors declare that the research was conducted in the absence of any commercial or financial relationships that could be construed as a potential conflict of interest.
