# Supplementary material for: Streptomyces antioxidans sp. nov., a Novel Mangrove Soil Actinobacterium with Antioxidative and Neuroprotective Potentials
Source: Front Microbiol. 2016 Jun 16;7:899. doi: 10.3389/fmicb.2016.00899 (PMC4909769; doi:10.3389/fmicb.2016.00899)
Supplement: Supplementary file 1 [file DataSheet1.pdf]

Fig. S1. Maximum-likelihood tree based on almost complete 16S rRNA sequences (1491 nucleotides) showing relationship between strain MUSC 164^{T} and representatives of some other related taxa. Bootstrap values (>50%) based on 1000 re-sampled datasets are shown at branch nodes. Bar, 0.002 substitutions per site. Asterisks indicate that the corresponding nodes were also recovered using neighbour-joining tree-making algorithms.

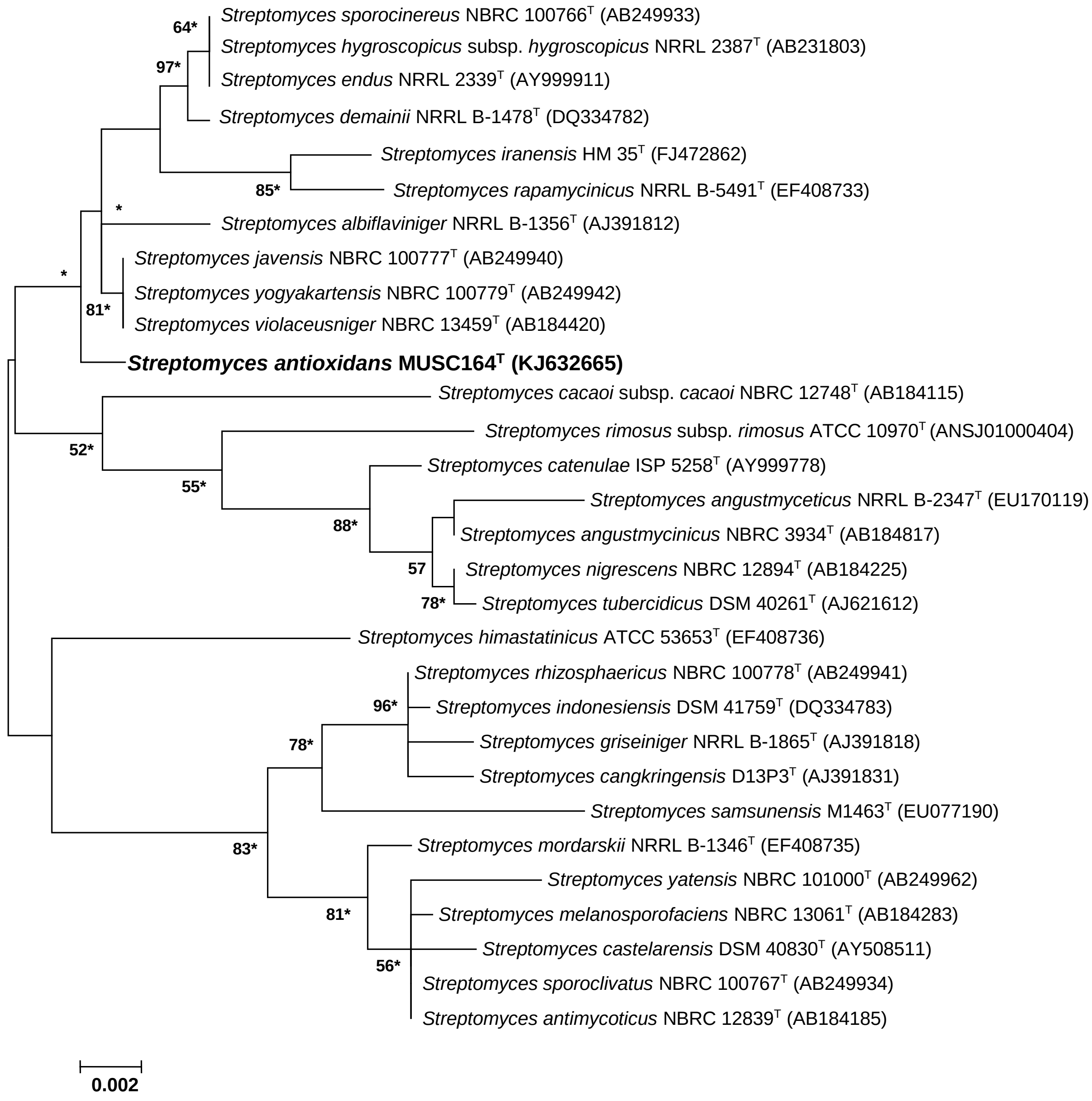

Fig. S2. BOX-PCR comparison of strain MUSC 164^T and the closest related type strains.

Lanes: 1, *Streptomyces antioxidans* sp. nov. MUSC 164^T; 2, *Streptomyces javensis*; 3, *Streptomyces violaceusniger*; 4, *Streptomyces yogyakartensis*; M, GeneRuler 1kb DNA ladder marker.

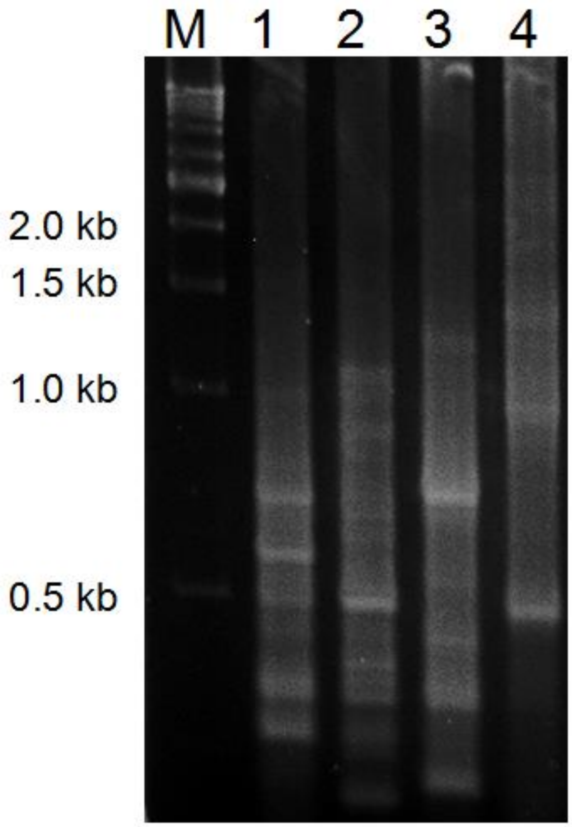

Fig. S3. Two dimensional total lipid profile of strain MUSC 164^T and *Streptomyces javensis* NBRC 100777^T.

AL, Aminolipid; DPG, Diphosphatidylglycerol; GL, Glycolipid; OH-PE, Hydroxyphosphatidylethanolamine; PL, Phospholipid; PI, Phosphatidylinositol; PE, Phosphatidylethanolamine; PG, Phosphatidylglycerol; PGL, Phosphoglycolipid; L, Lipid.

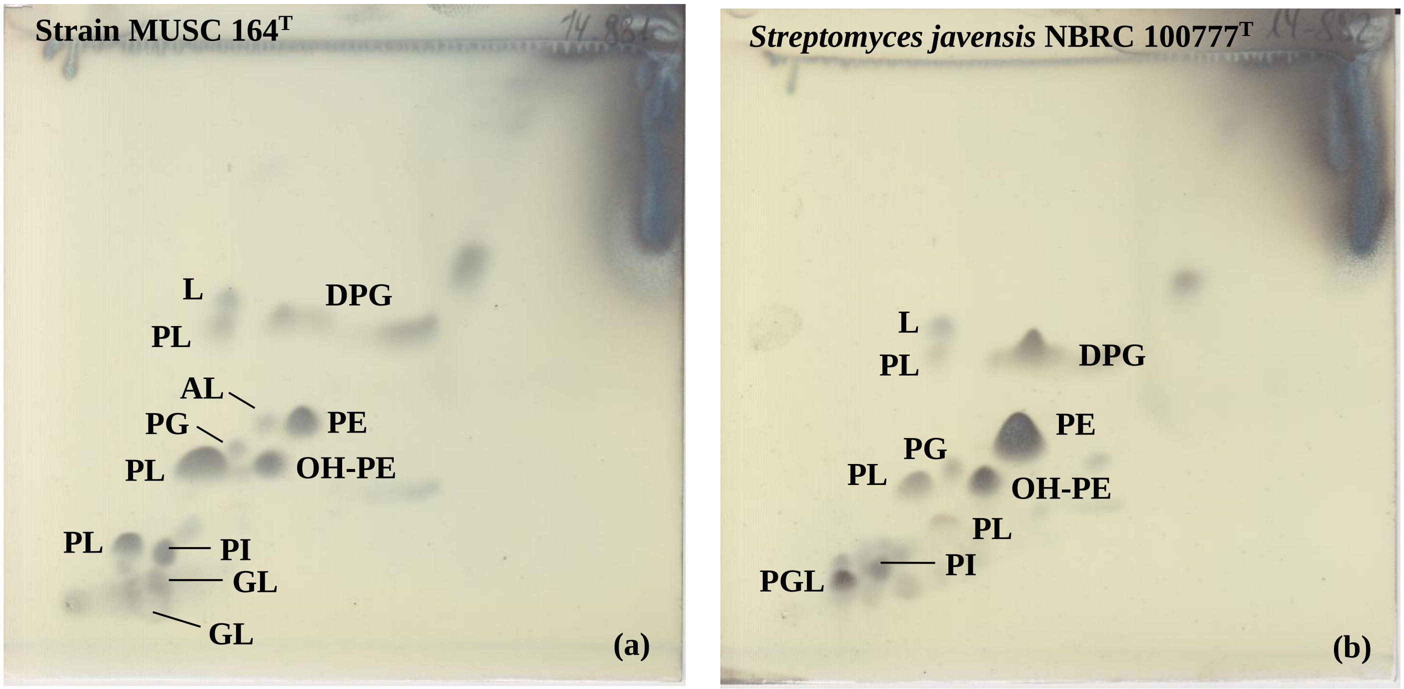

Fig. S4. The mass spectrum of the constituents (1-24) identified from the GC-MS analysis. (a) The mass spectrum of the constituents obtained from the MUSC 164[T], (b) the mass spectrum of the standard compounds available on NIST05 Spectral Library.

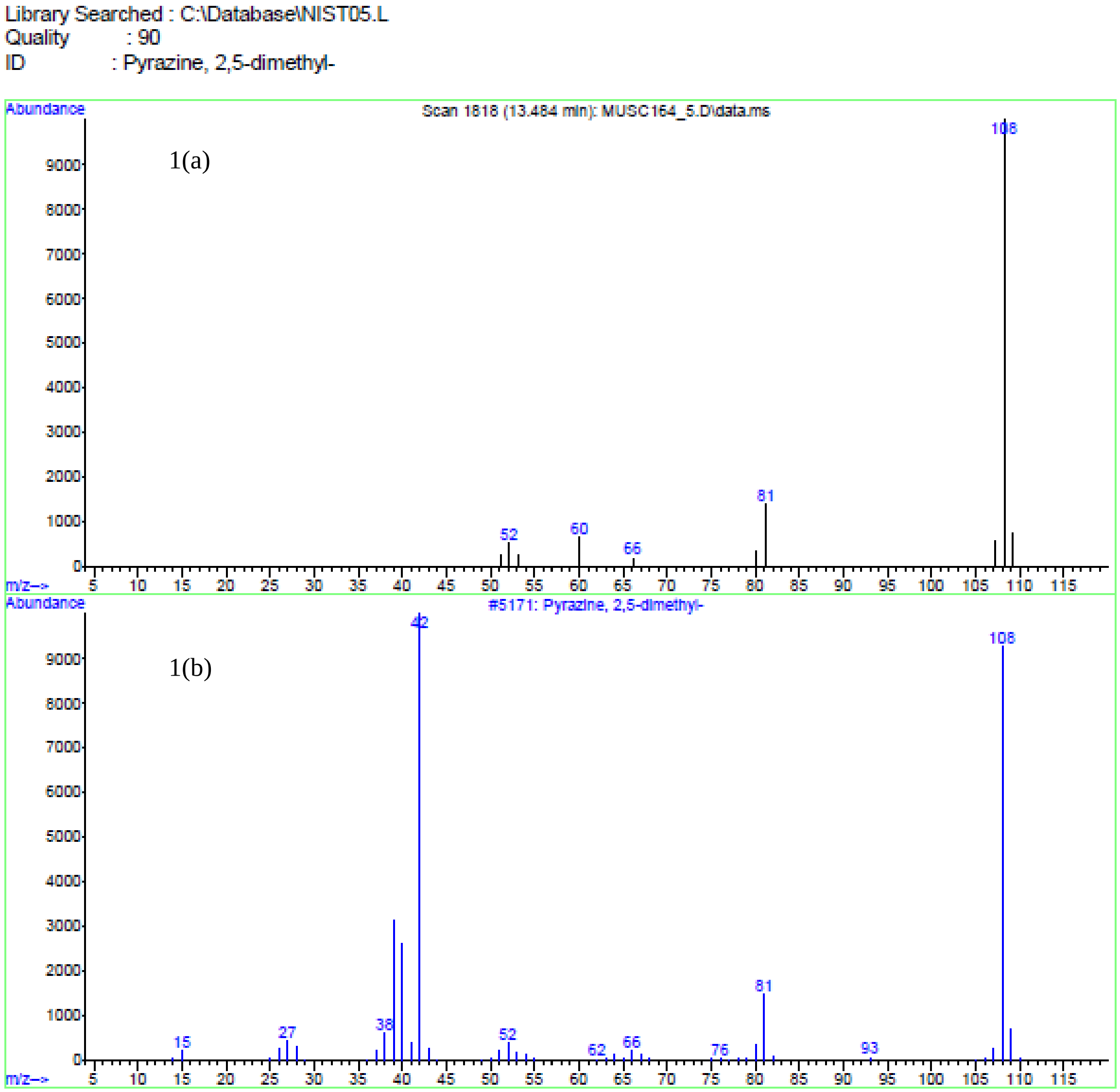

Library Searched : C:\Database\NIST05.L
Quality : 80
ID : Pyrazine, 2,3-dimethyl-

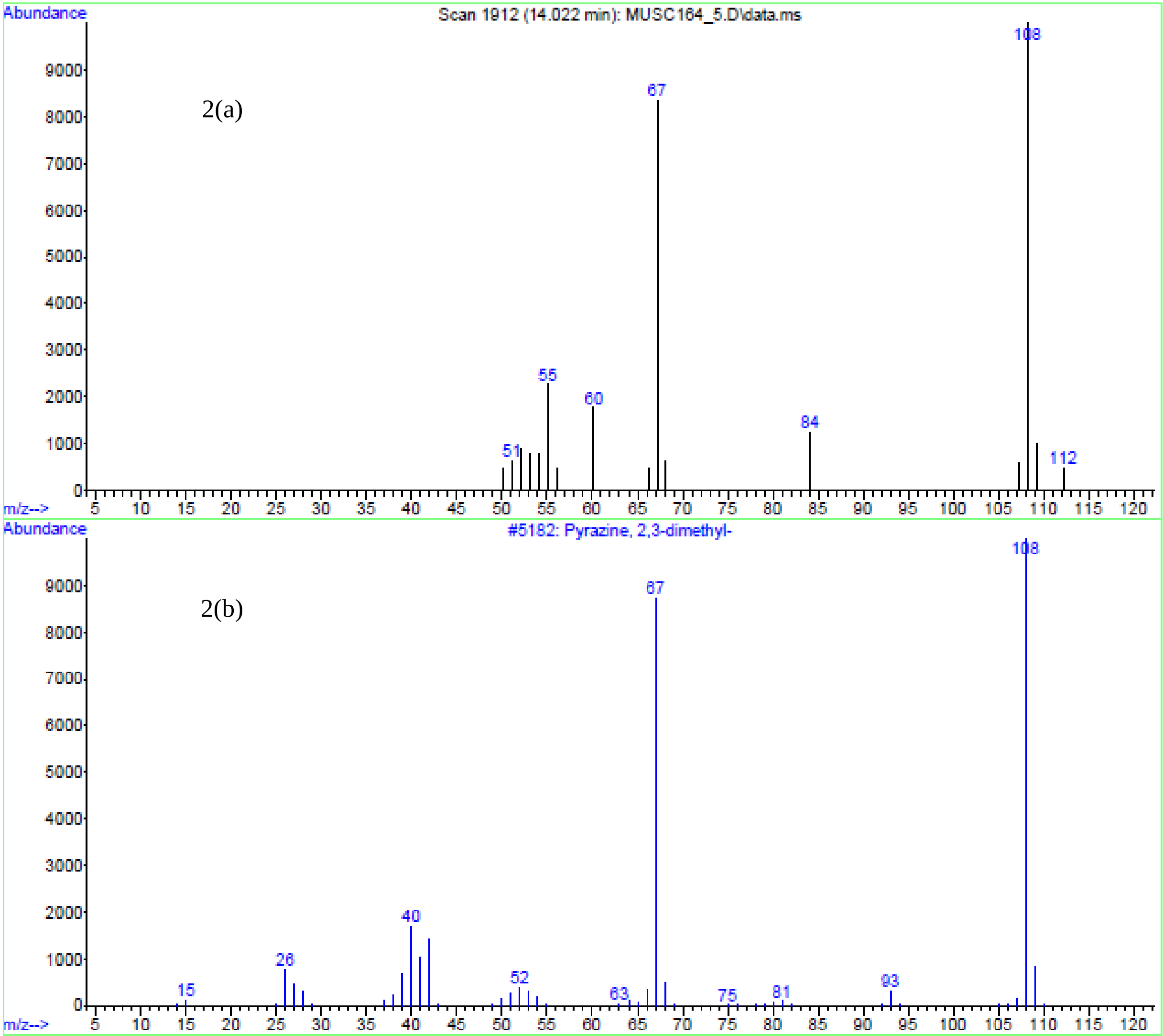

Library Searched : C:\Database\NIST05.L
Quality : 78
ID : Dimethyl trisulfide

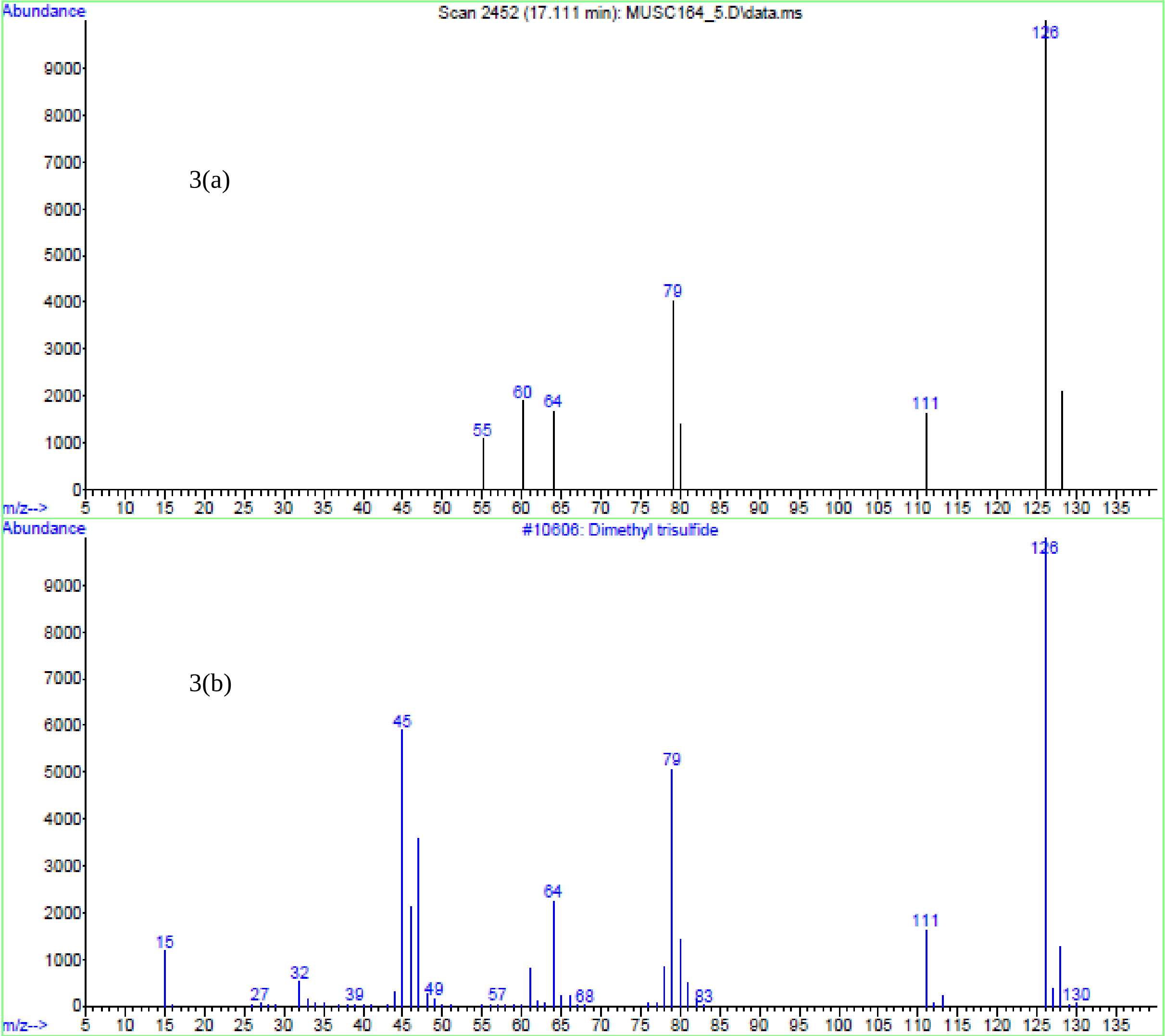

Library Searched : C:\Database\NIST05.L
Quality : 90
ID : Pyrazine, 2-ethyl-5-methyl-

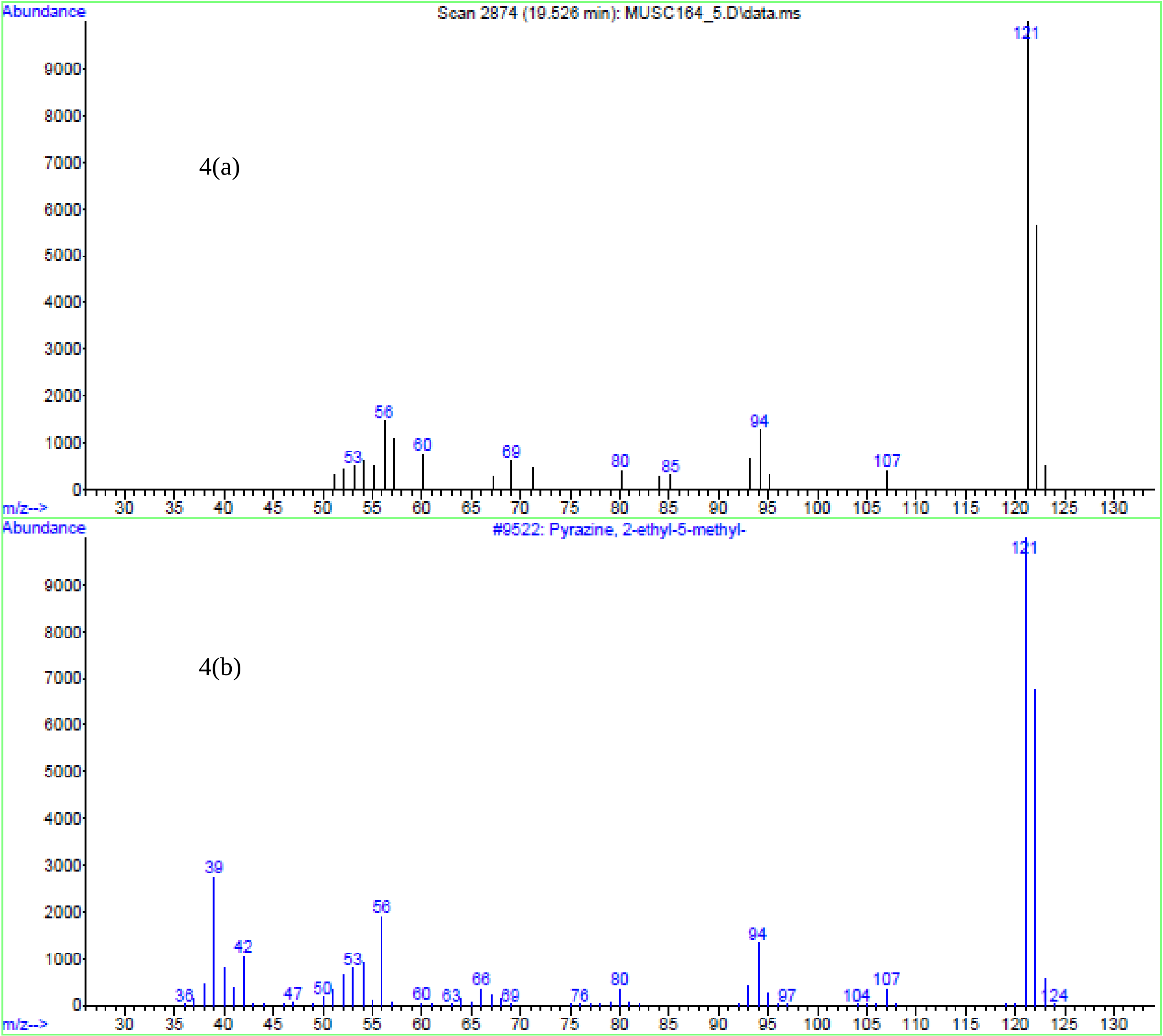

76

Library Searched : C:\Database\NIST05.L
Quality : 78
ID : Pyrazine, trimethyl-

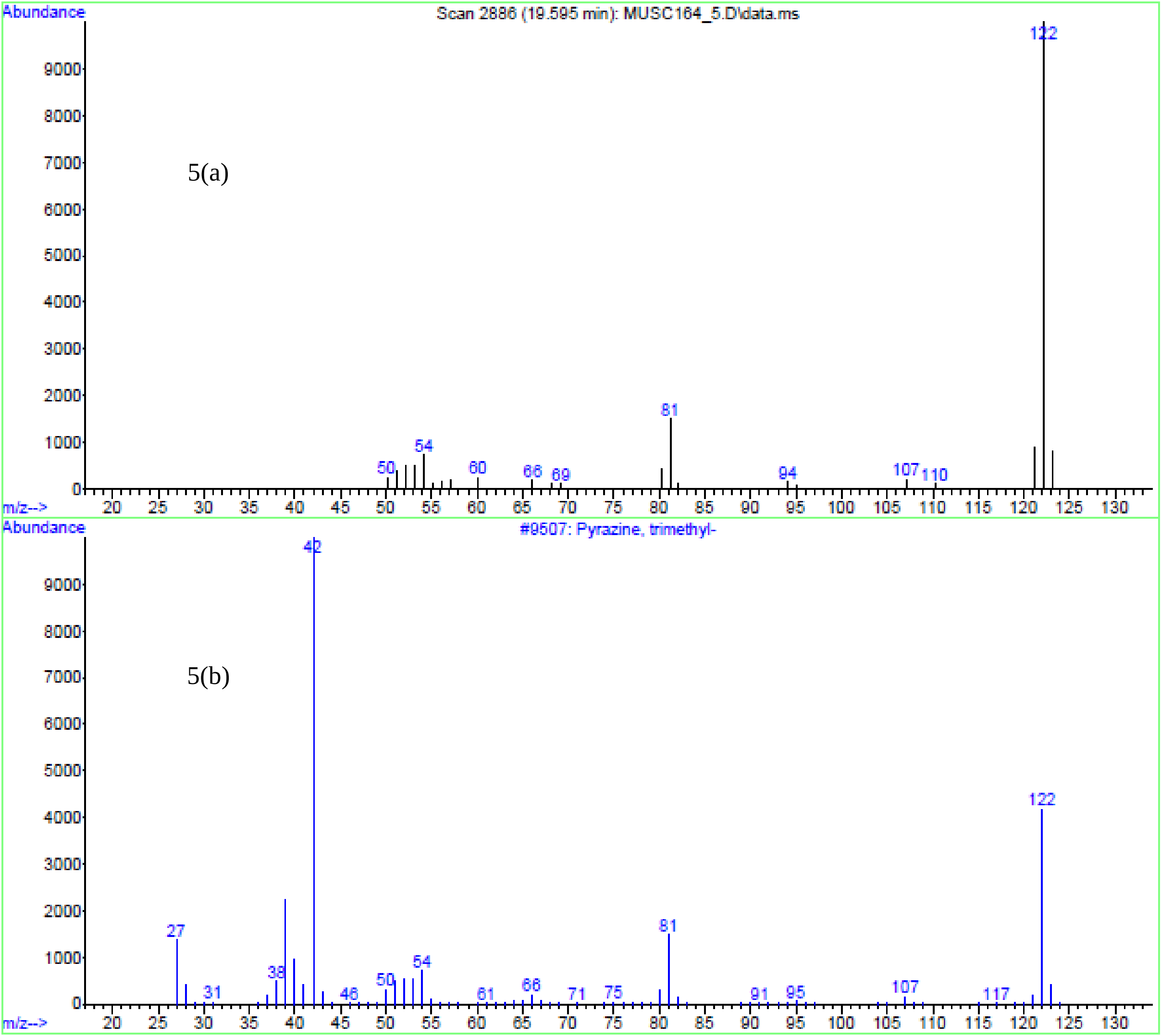

Library Searched : C:\Database\NIST05.L
Quality : 94
ID : Pyrazine, 3-ethyl-2,5-dimethyl-

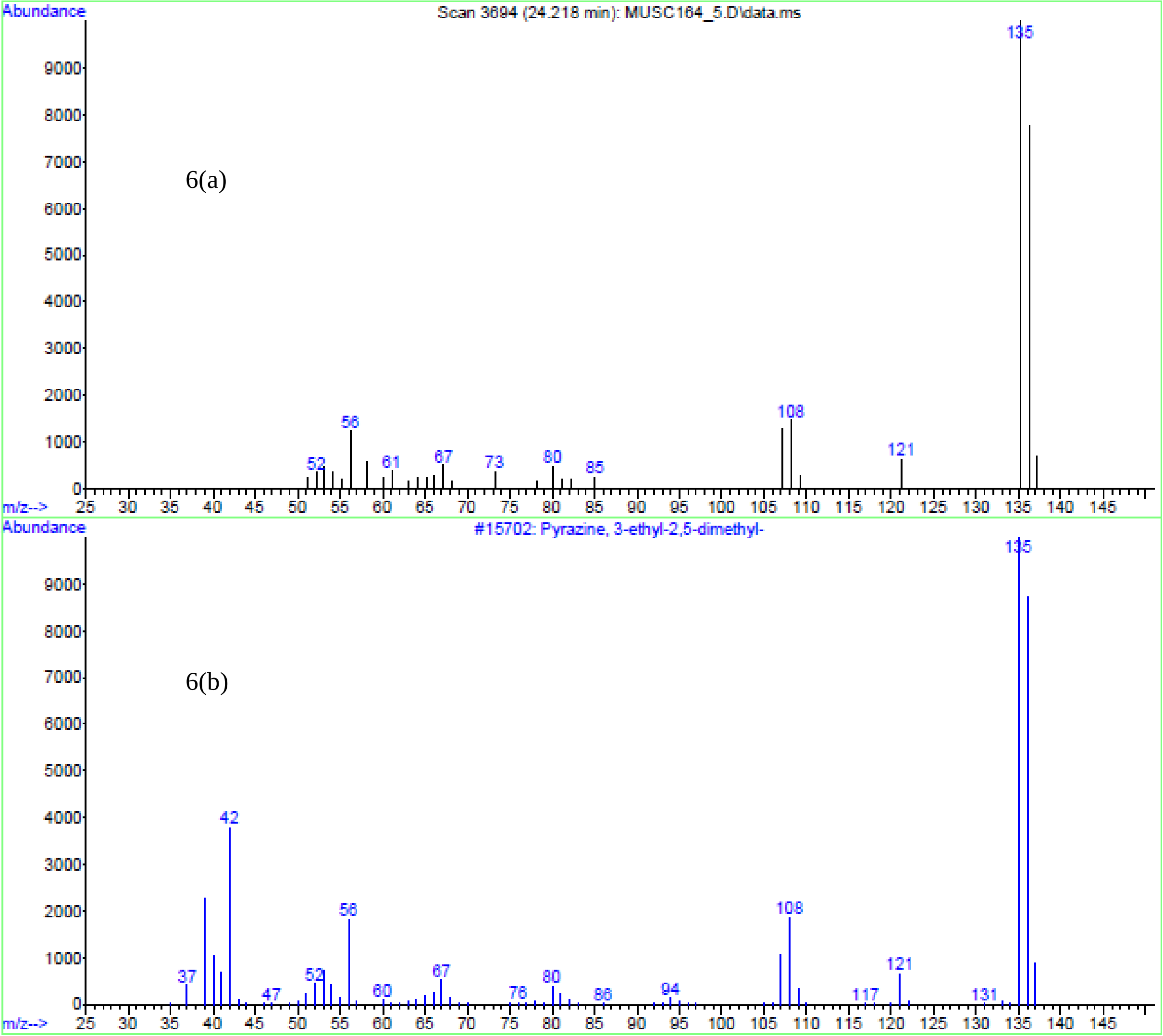

Library Searched : C:\Database\NIST05.L
Quality : 53
ID : 4-Pyridinamine, N,N,2-trimethyl-

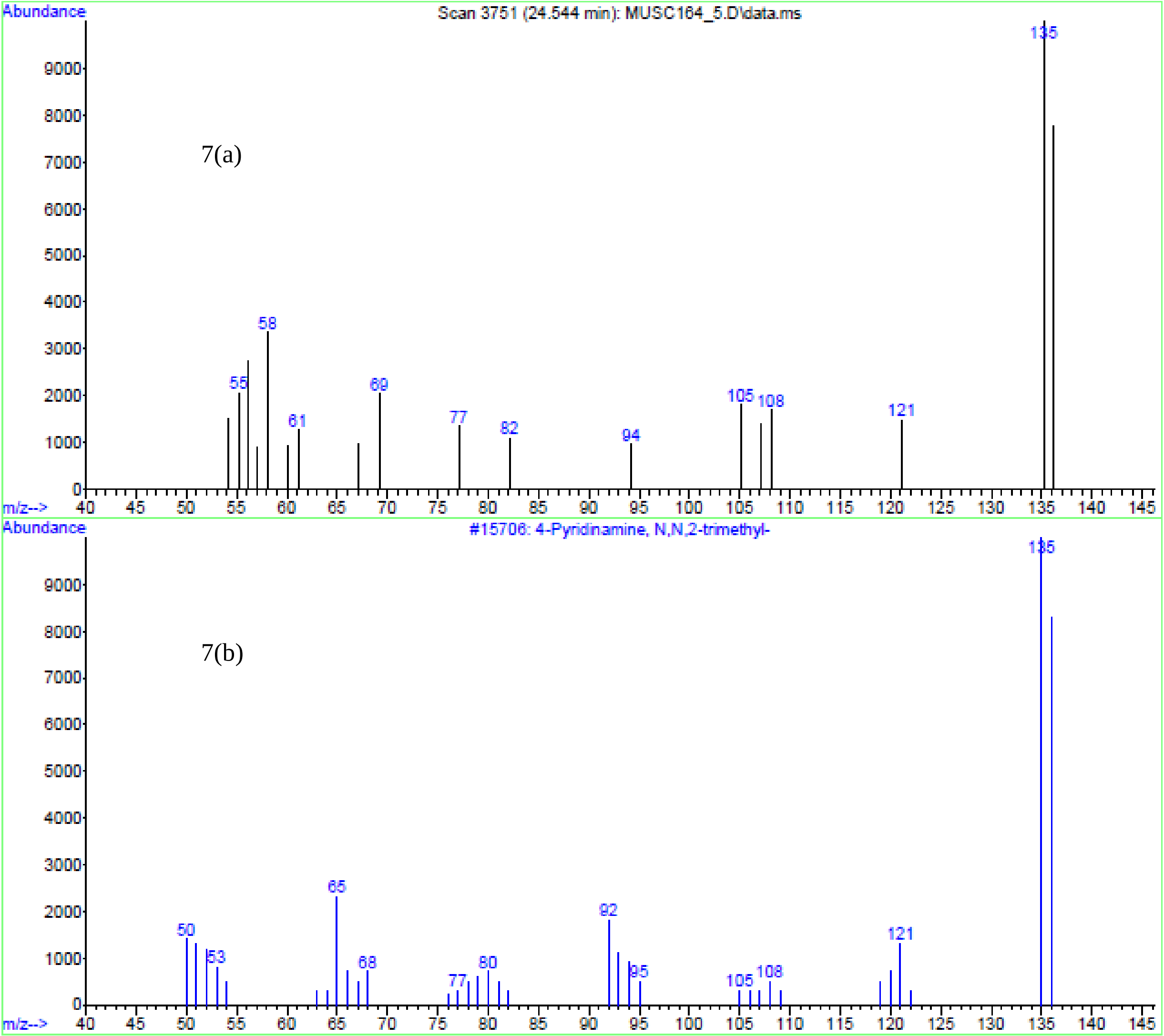

Library Searched : C:\Database\NIST05.L
Quality : 91
ID : 2,3-Dimethyl-5-ethylpyrazine

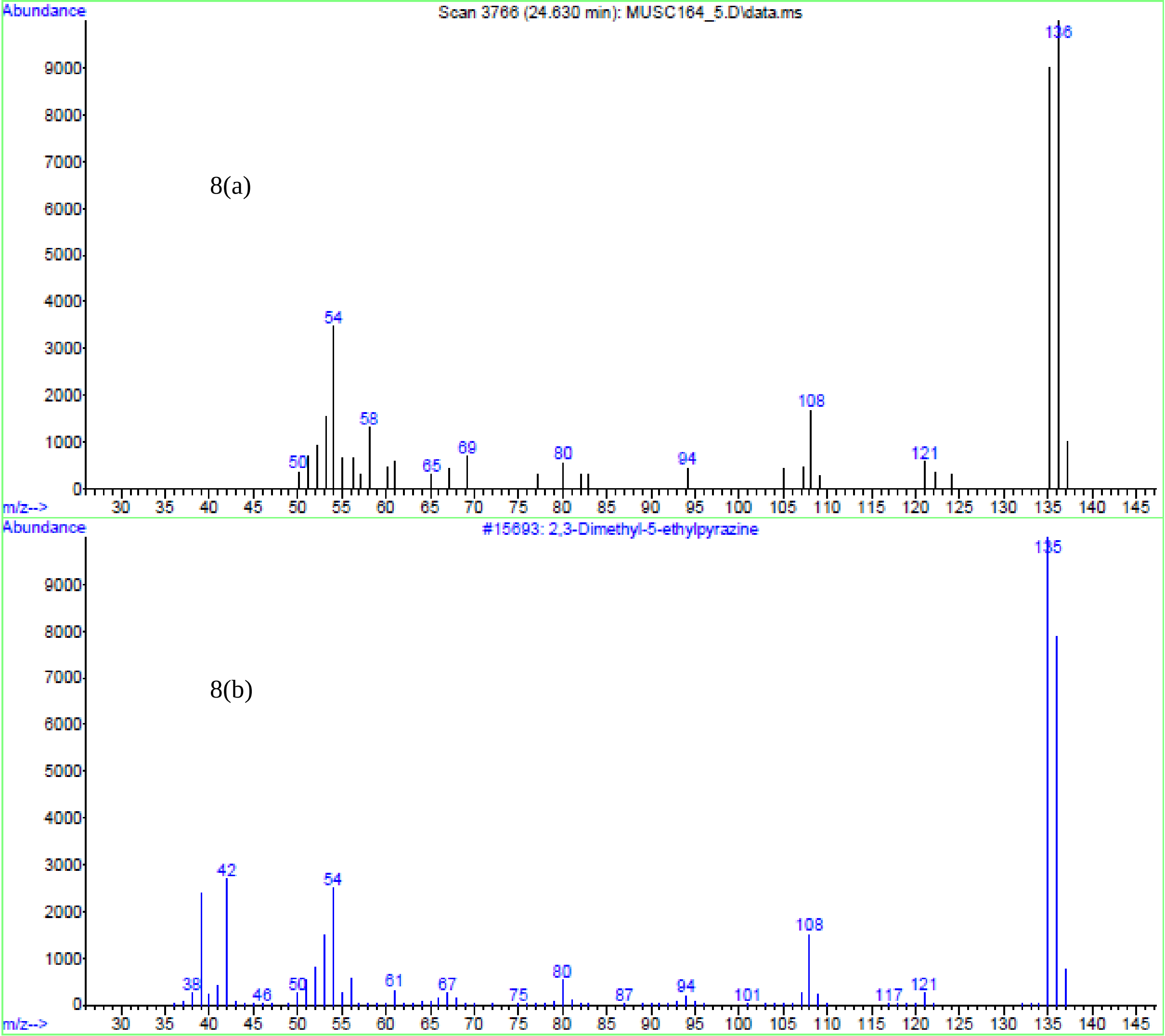

Library Searched : C:\Database\NIST05.L
Quality : 90
ID : Benzoic acid, methyl ester

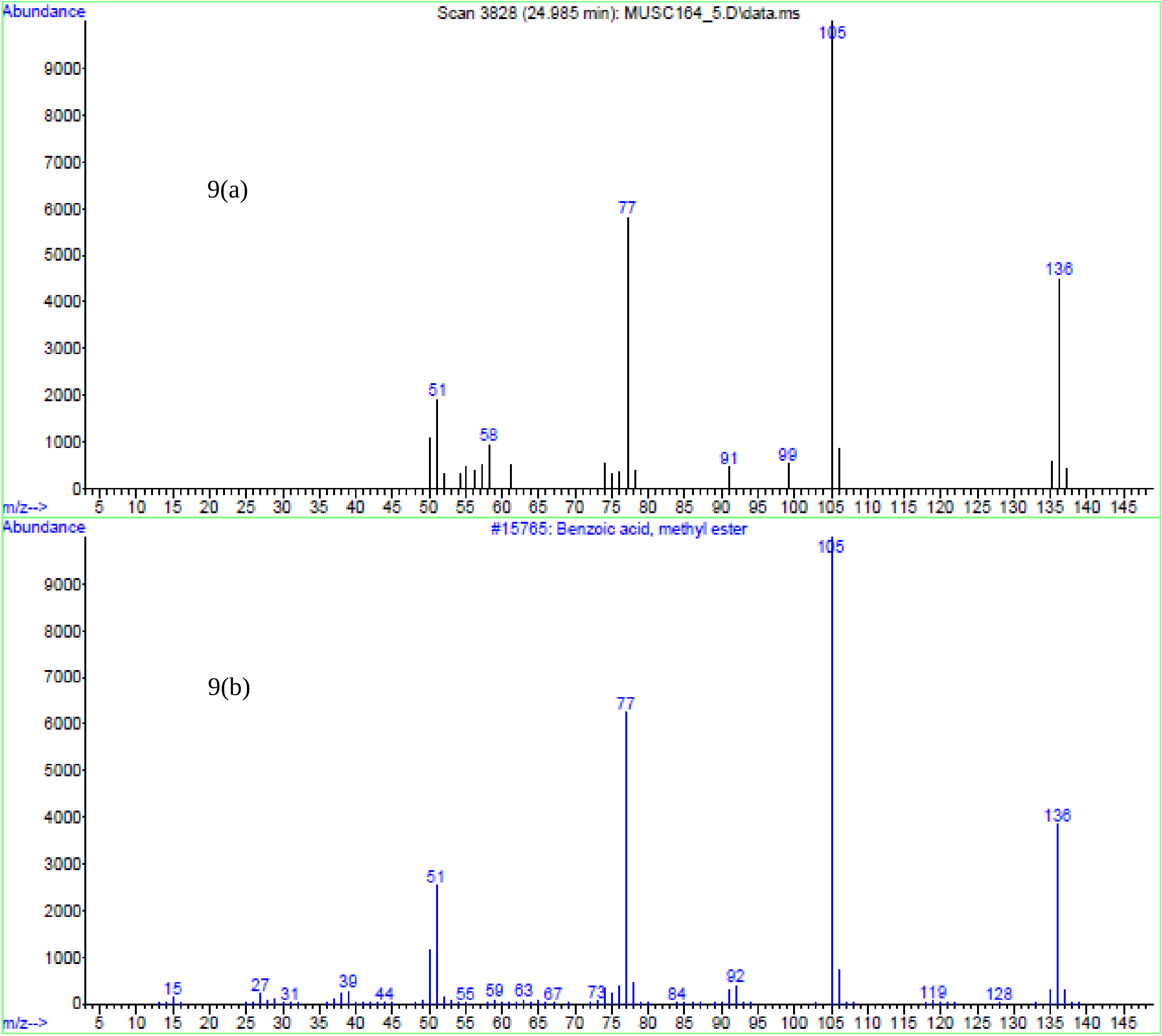

Library Searched : C:\Database\NIST05.L
Quality : 59
ID : Pyrazine, 2-methyl-5-(1-propenyl)-, (E)-

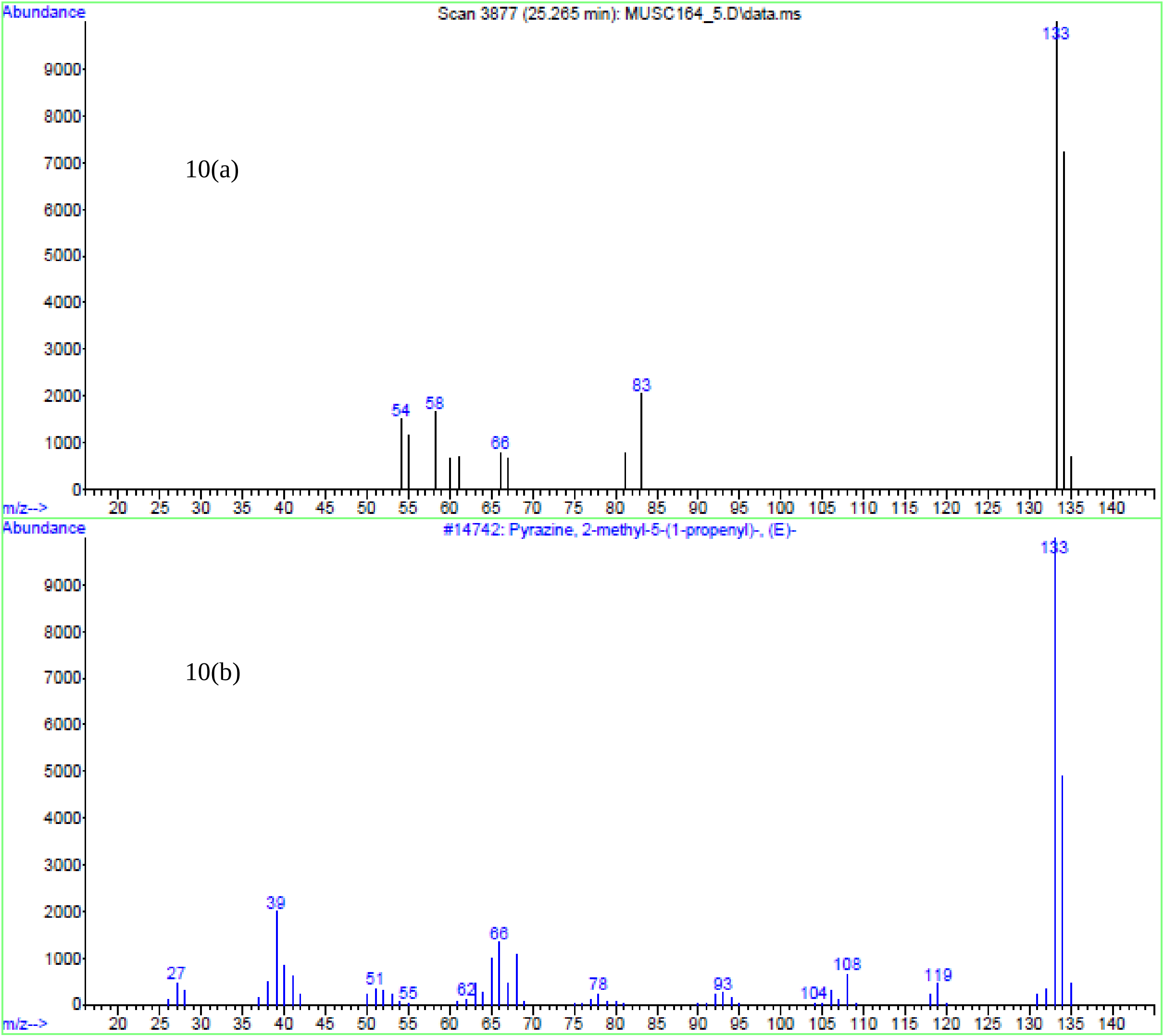

89

Library Searched : C:\Database\NIST05.L
Quality : 72
ID : Pyrazine, 3,5-diethyl-2-methyl-

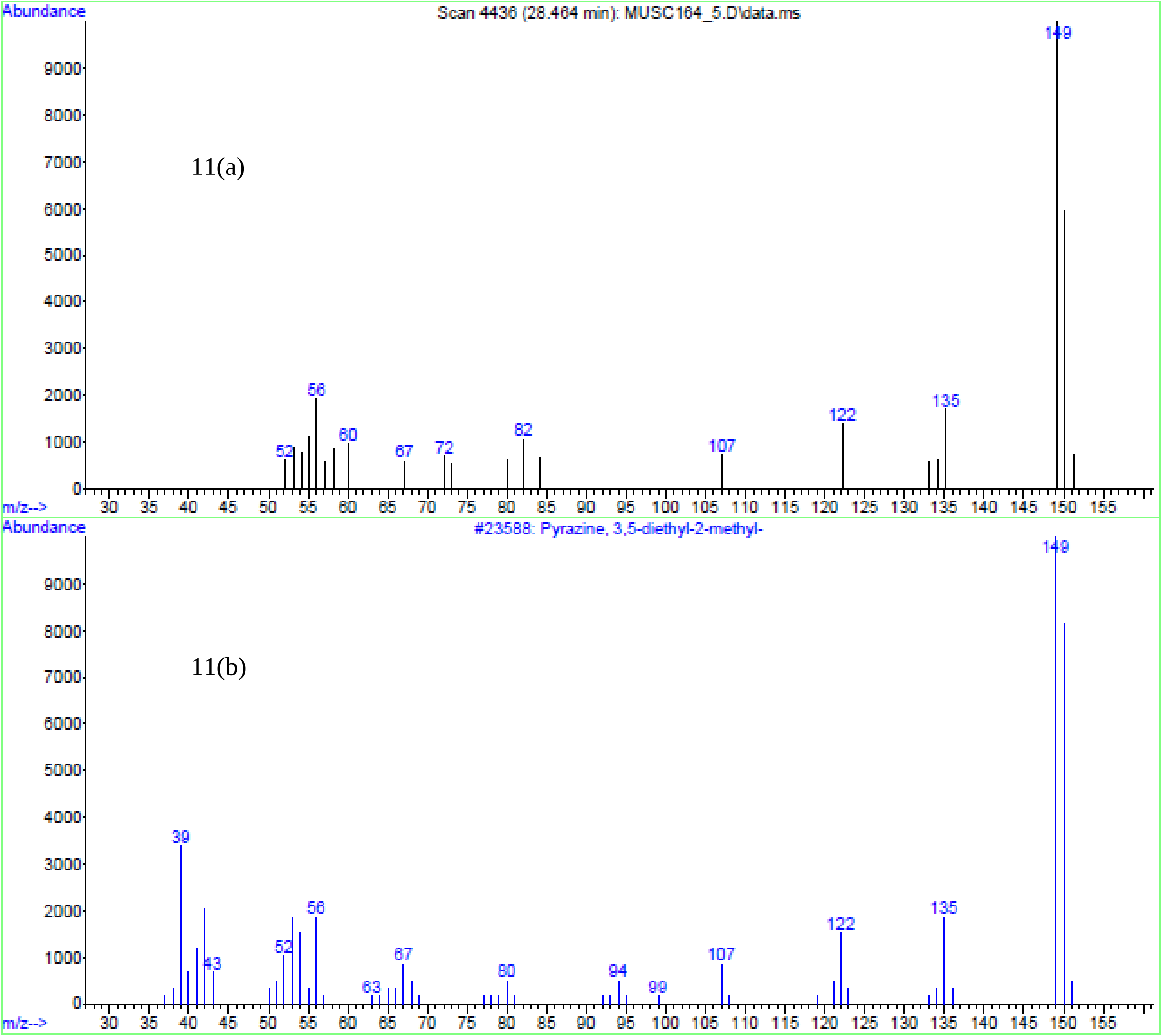

Library Searched : C:\Database\NIST05.L
Quality : 59
ID : 2-Piperidinone

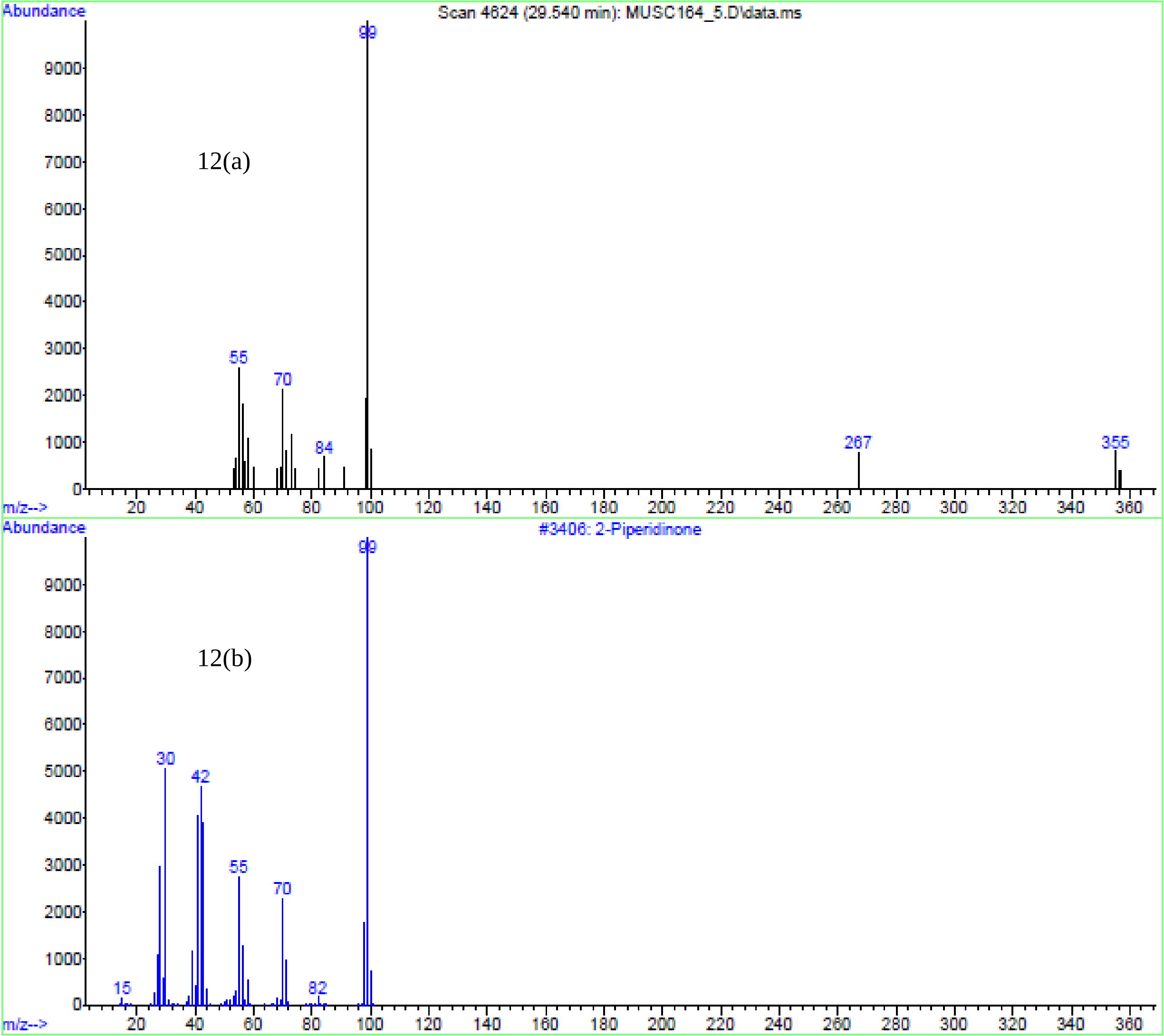

91

Library Searched : C:\Database\NIST05.L
Quality : 80
ID : Pyrazine, 2,5-dimethyl-3-(2-methylpropyl)-

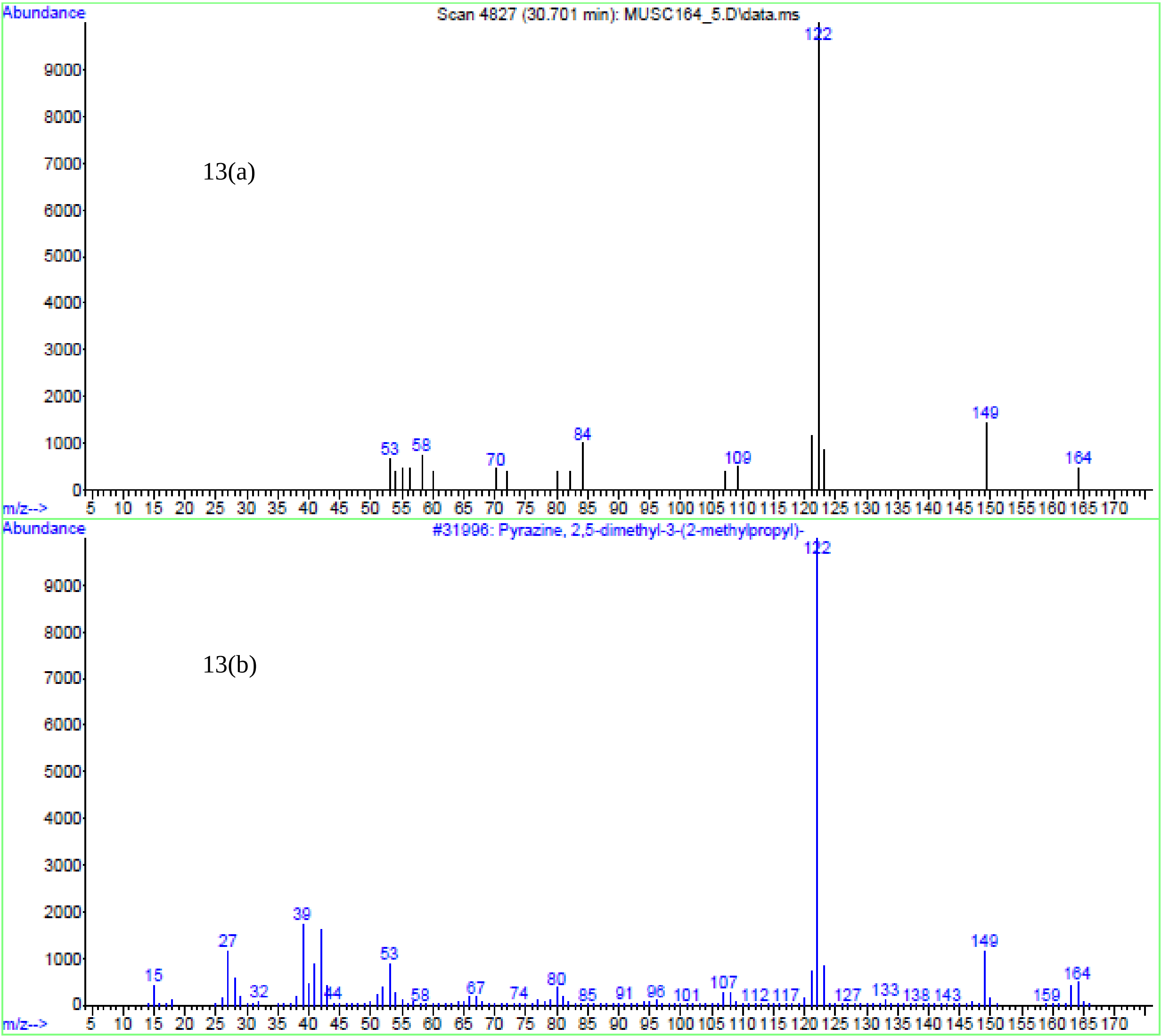

Library Searched : C:\Database\NIST05.L
Quality : 64
ID : Indolizine

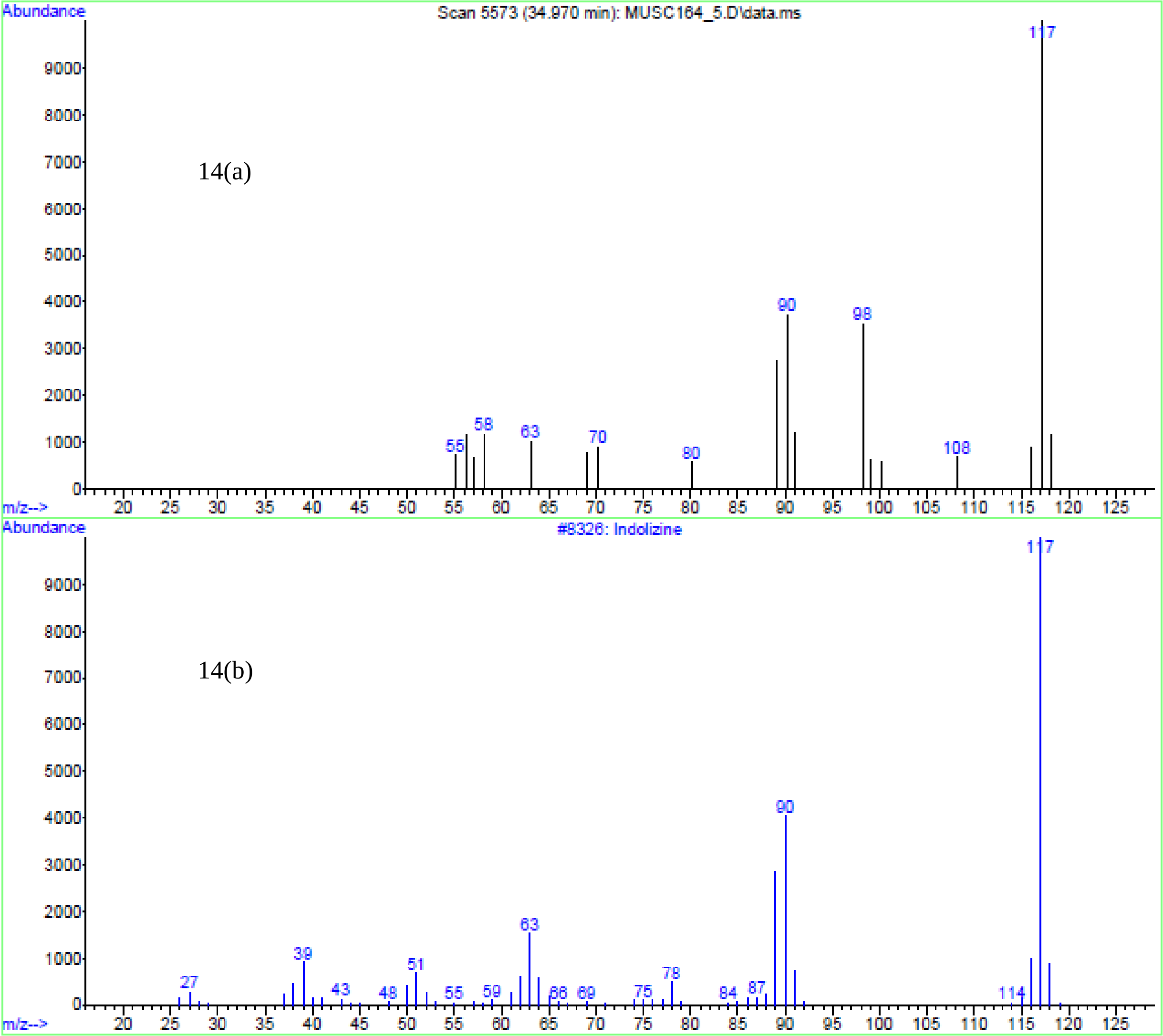

Library Searched : C:\Database\NIST05.L
Quality : 83
ID : Pyrazine, 2,5-dimethyl-3-(3-methylbutyl)-

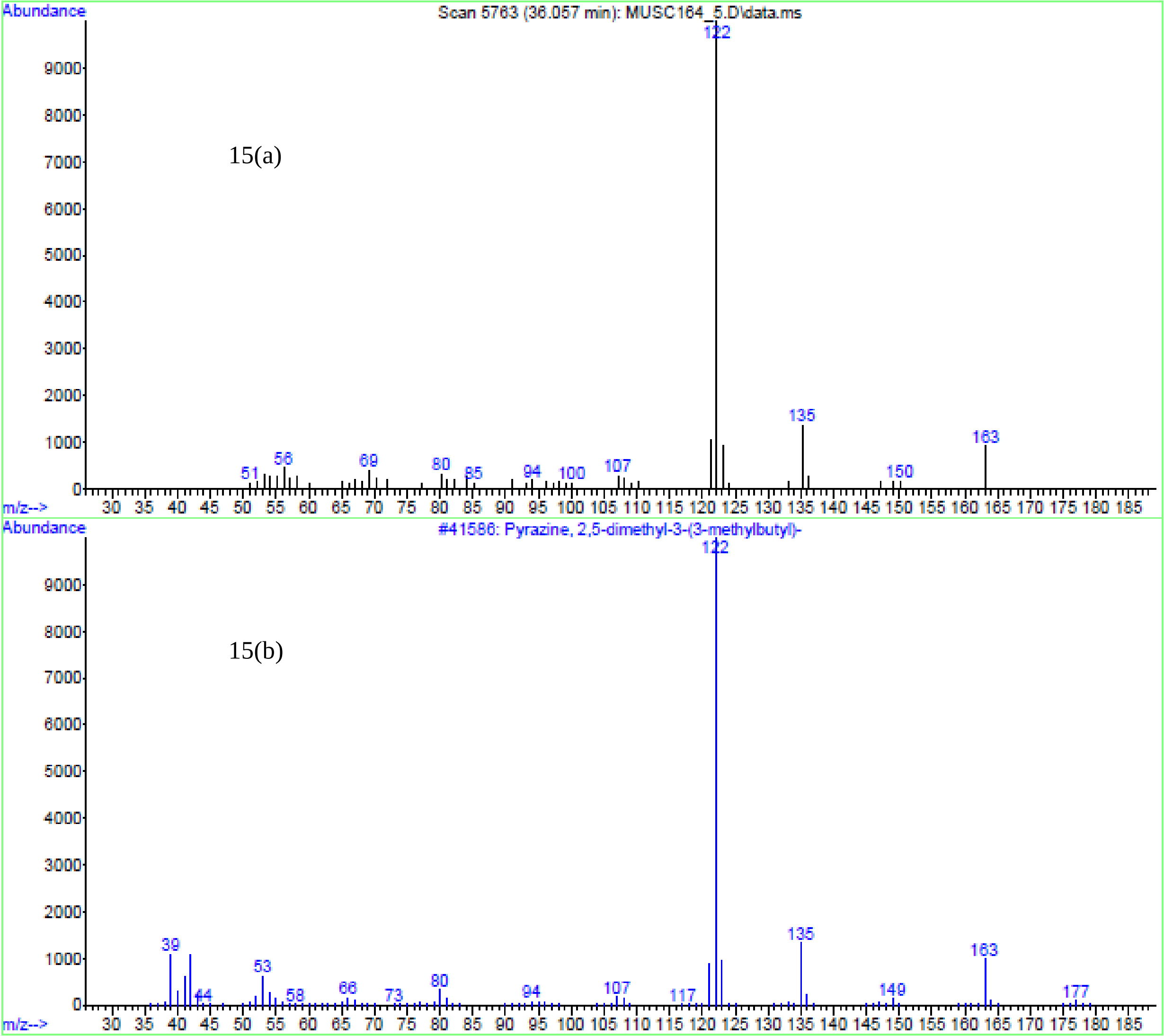

96

Library Searched : C:\Database\NIST05.L
Quality : 64
ID : Pyrazine, 3,5-dimethyl-2-propyl-

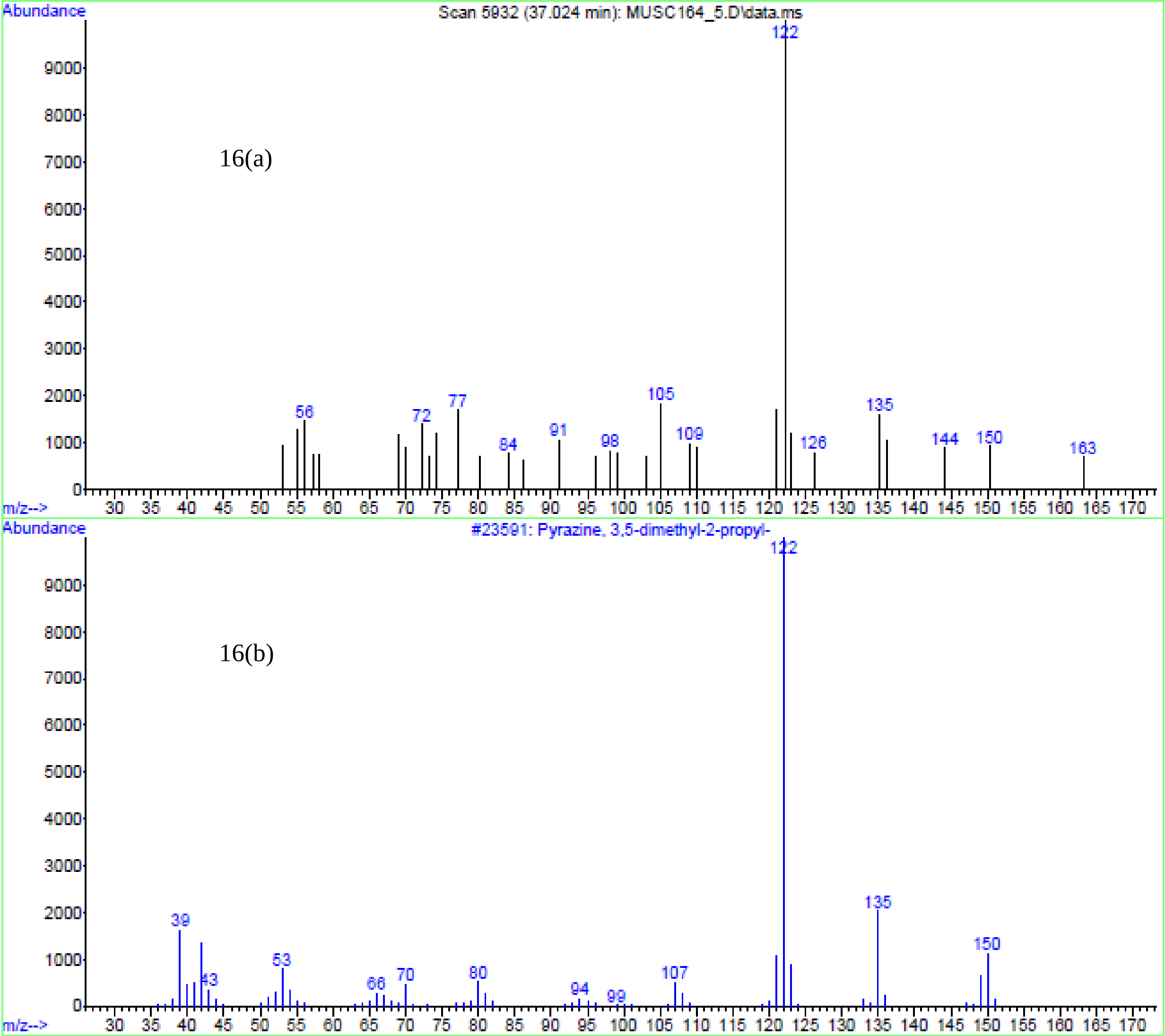

Library Searched : C:\Database\NIST05.L
Quality : 68
ID : 2,3,5-Trimethyl-6-ethylpyrazine

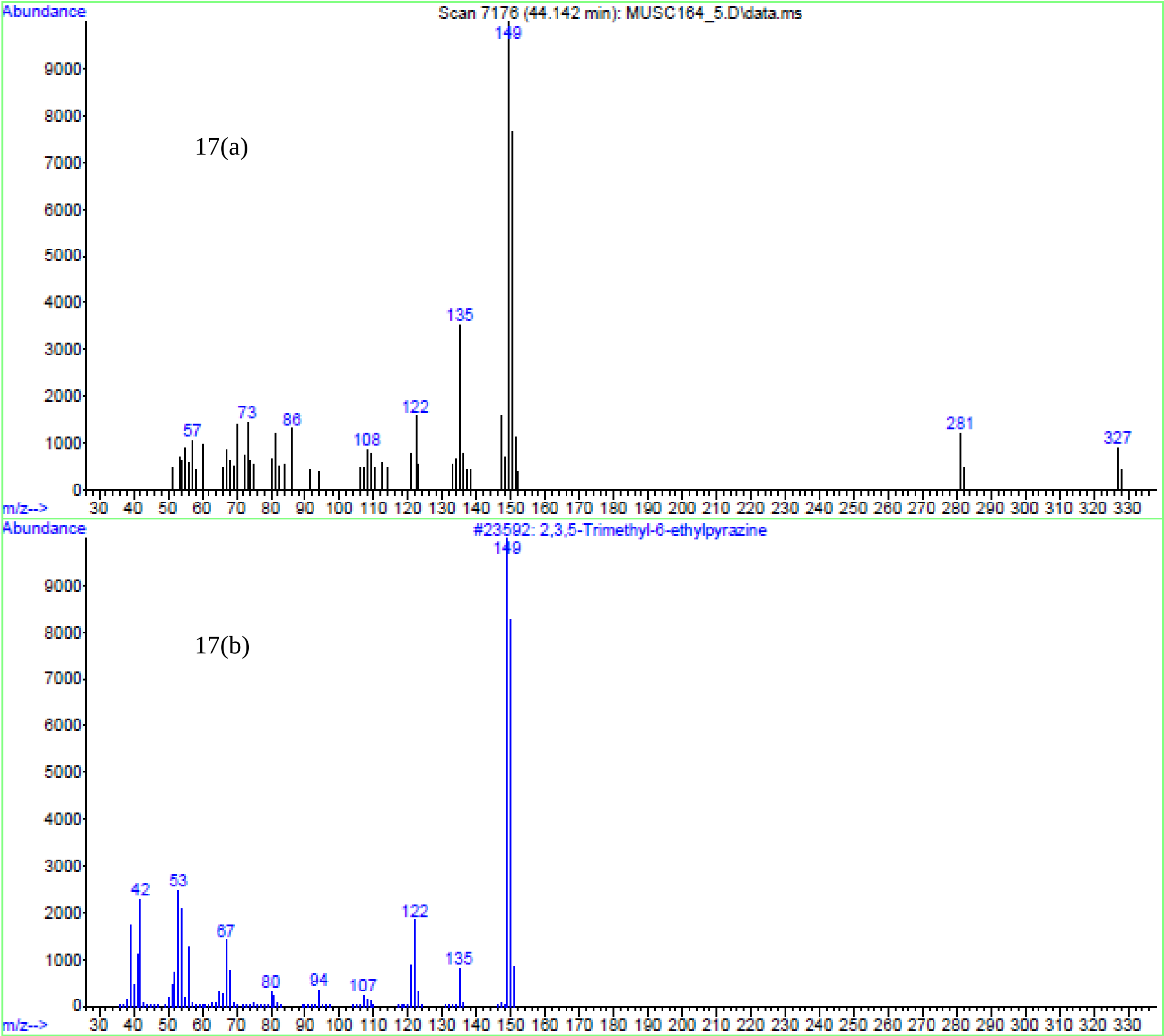

98

Library Searched : C:\Database\NIST05.L
Quality : 95
ID : Phenol, 2,4-bis(1,1-dimethylethyl)-

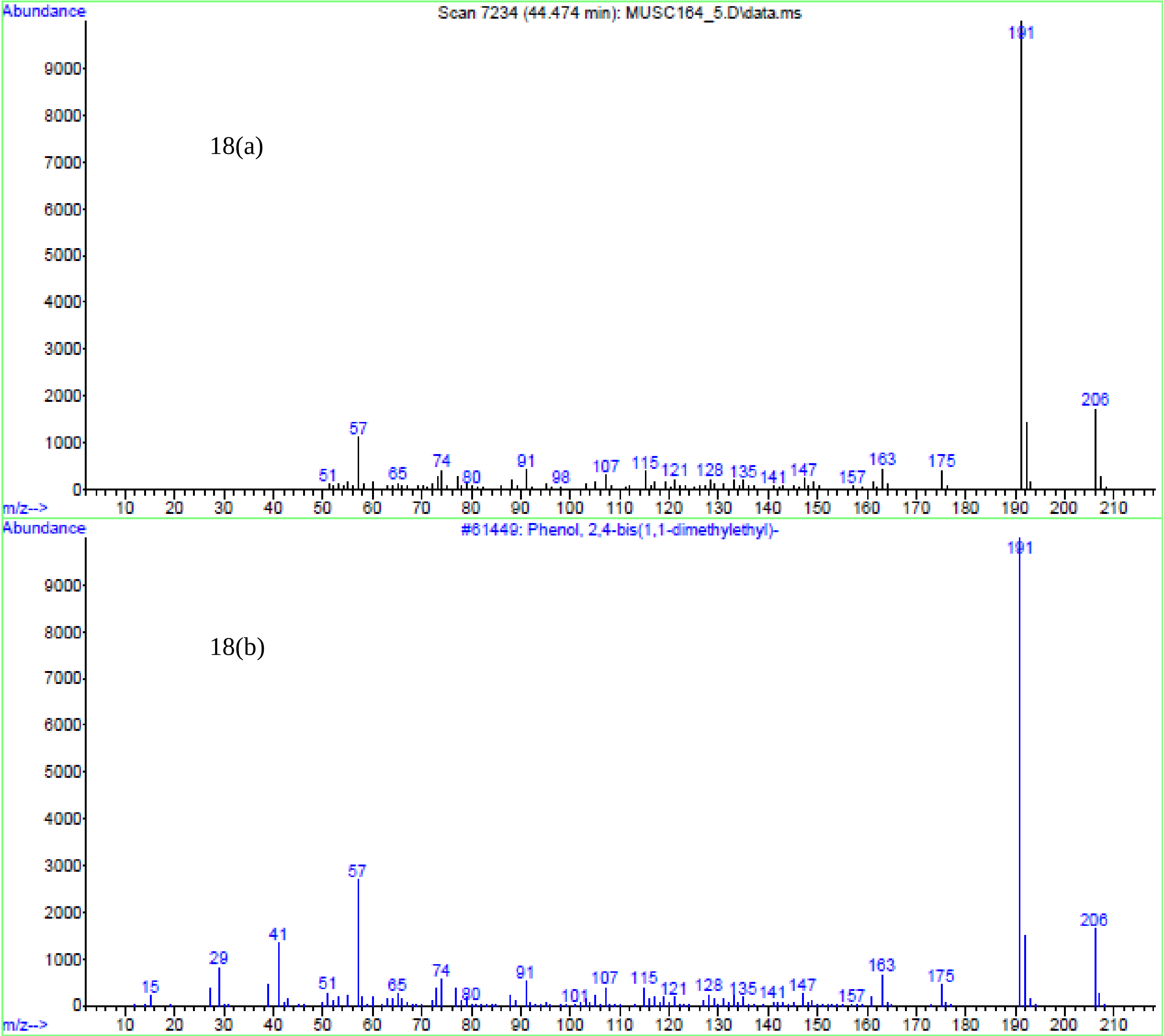

99

Library Searched : C:\Database\NIST05.L
Quality : 87
ID : 1,2,3,4-Tetrahydro-cyclopenta[b]indole

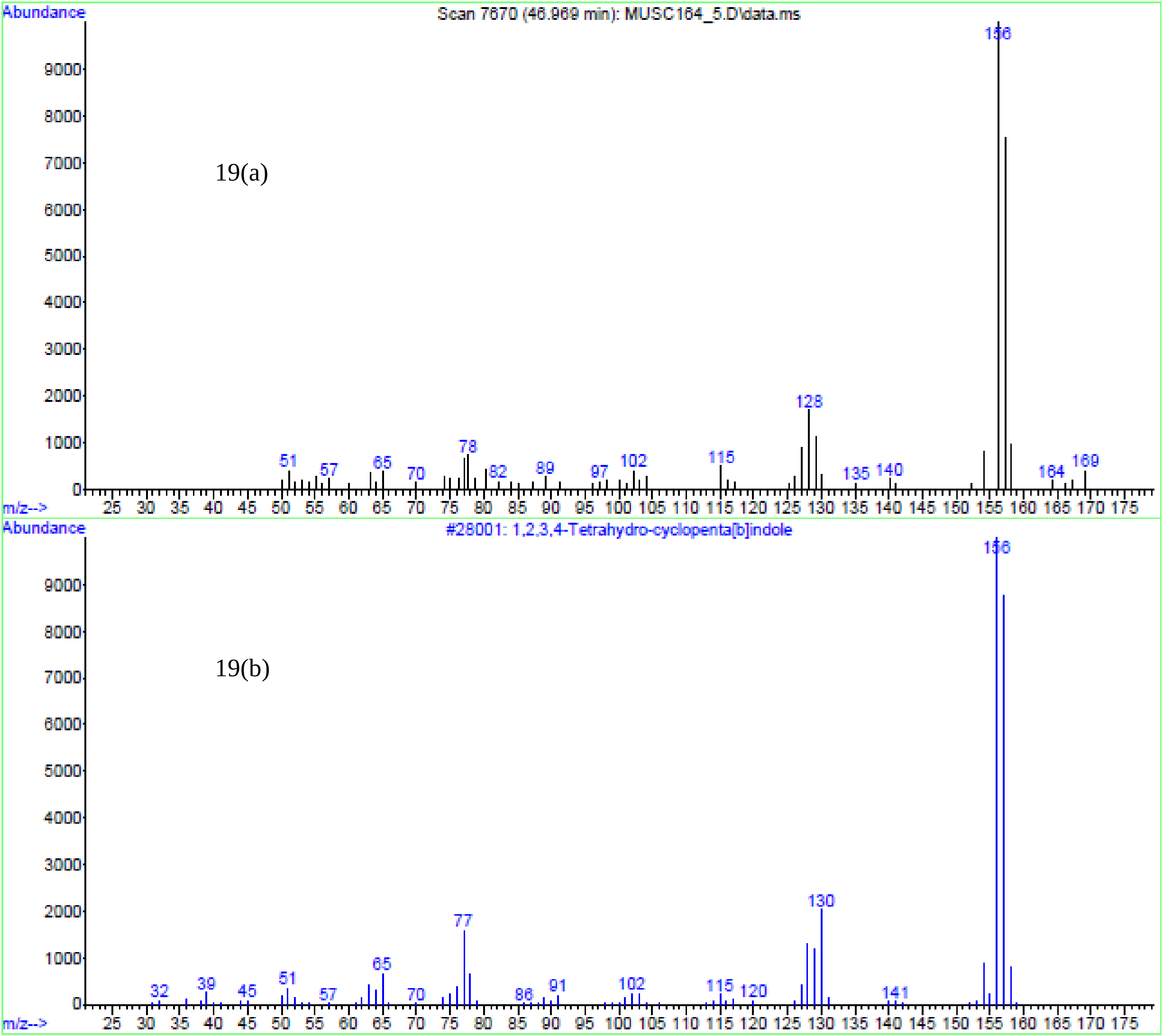

Library Searched : C:\Database\NIST05.L
Quality : 97
ID : Pyrrolo[1,2-a]pyrazine-1,4-dione, hexahydro-

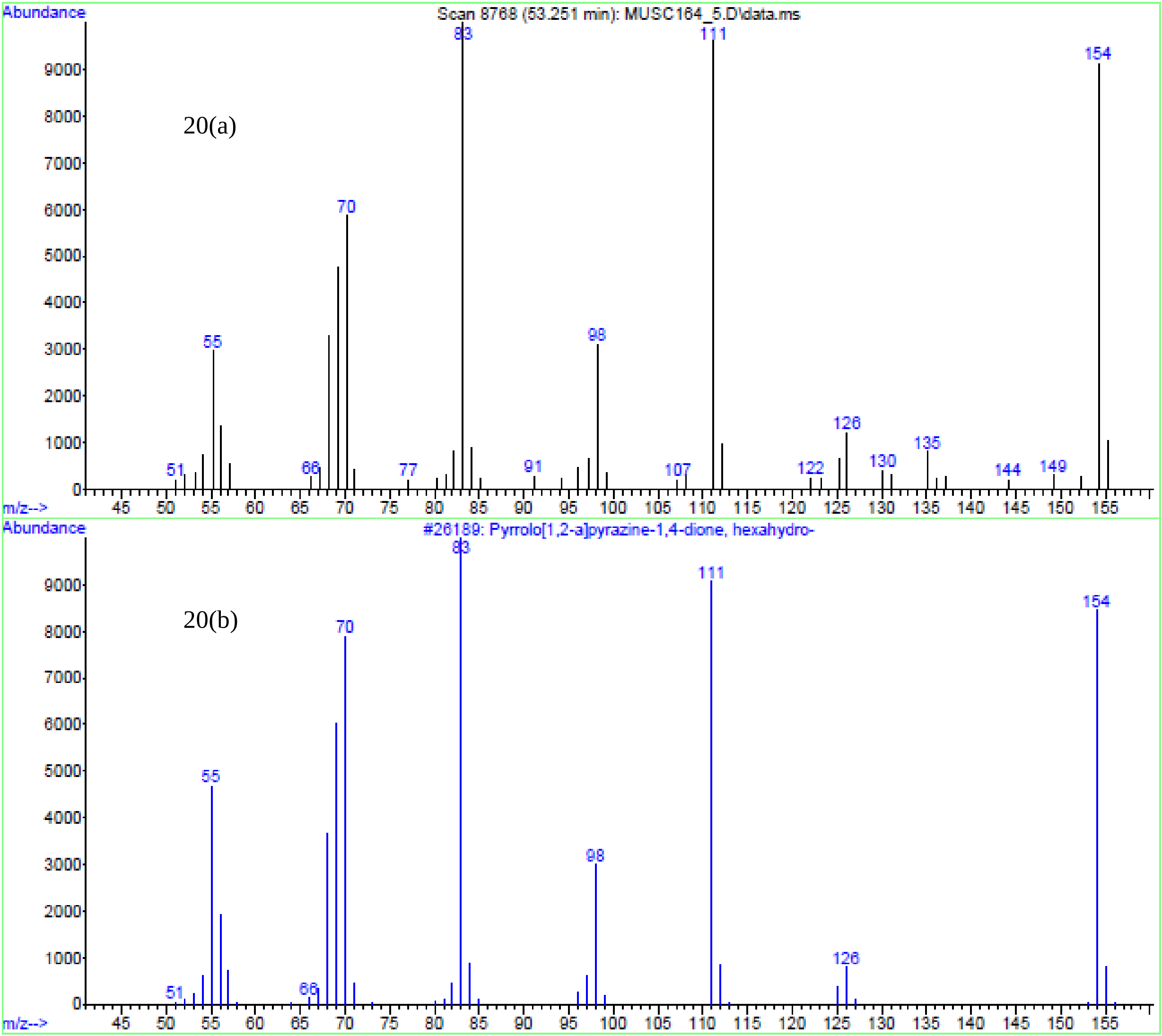

101

Library Searched : C:\Database\NIST05.L
Quality : 58
ID : Phenol, 3,5-dimethoxy-

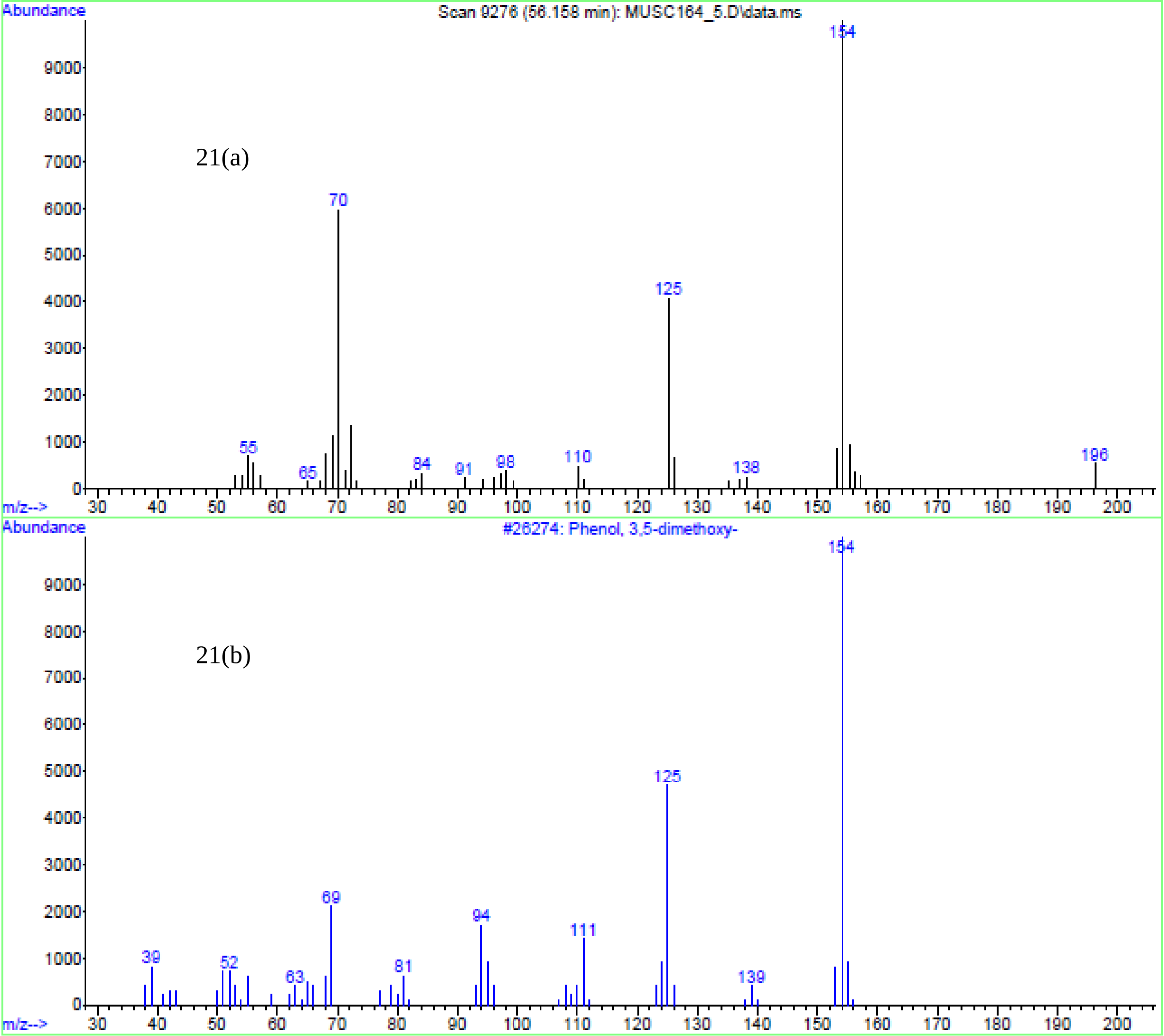

Library Searched : C:\Database\NIST05.L
Quality : 94
ID : Hexadecanoic acid, methyl ester

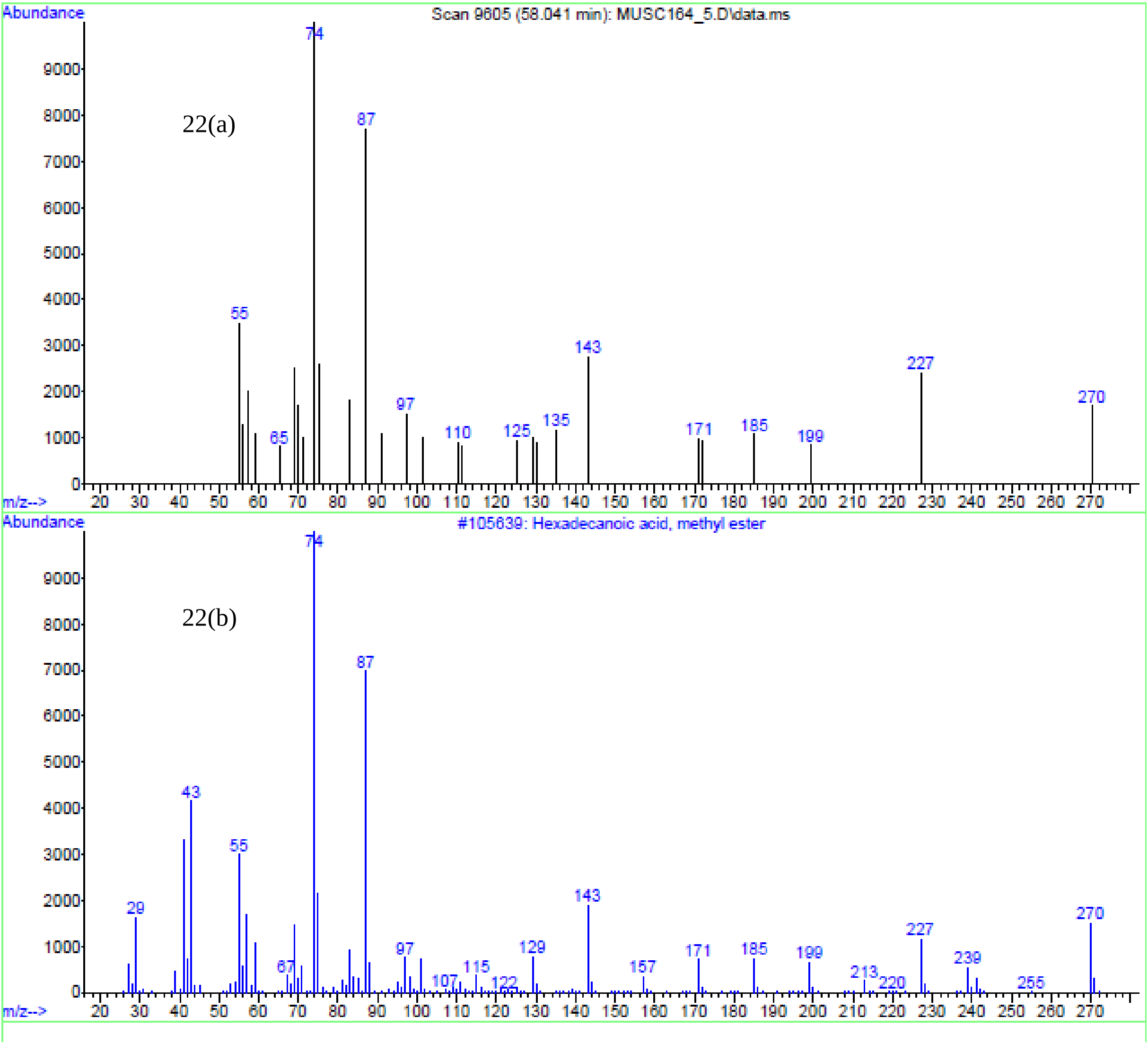

Library Searched : C:\Database\NIST05.L
Quality : 90
ID : Pentadecanoic acid, 14-methyl-, methyl ester

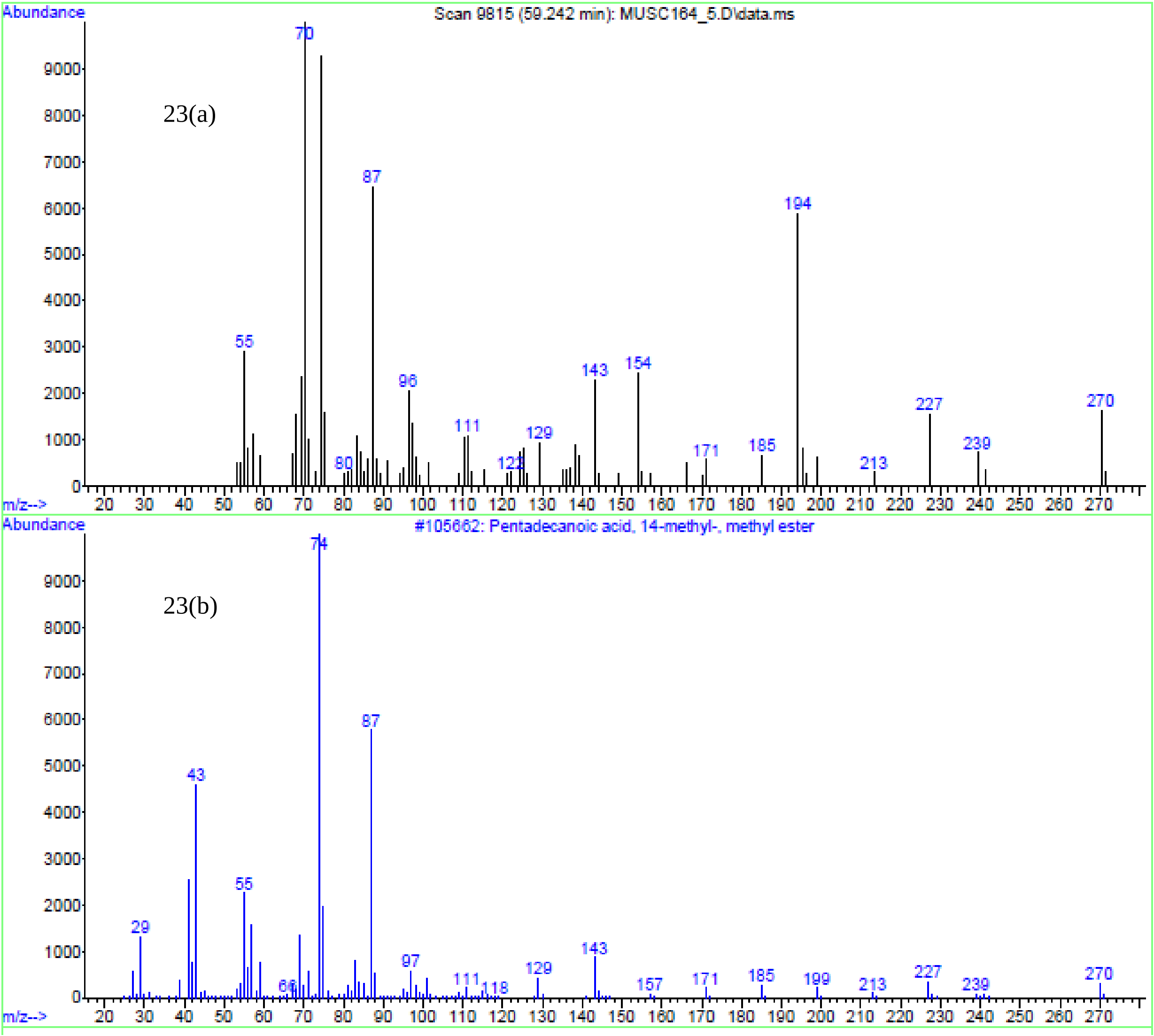

105

Library Searched : C:\Database\NIST05.L
Quality : 97
ID : Pyrrolo[1,2-a]pyrazine-1,4-dione, hexahydro-3-(phenylmethyl)-

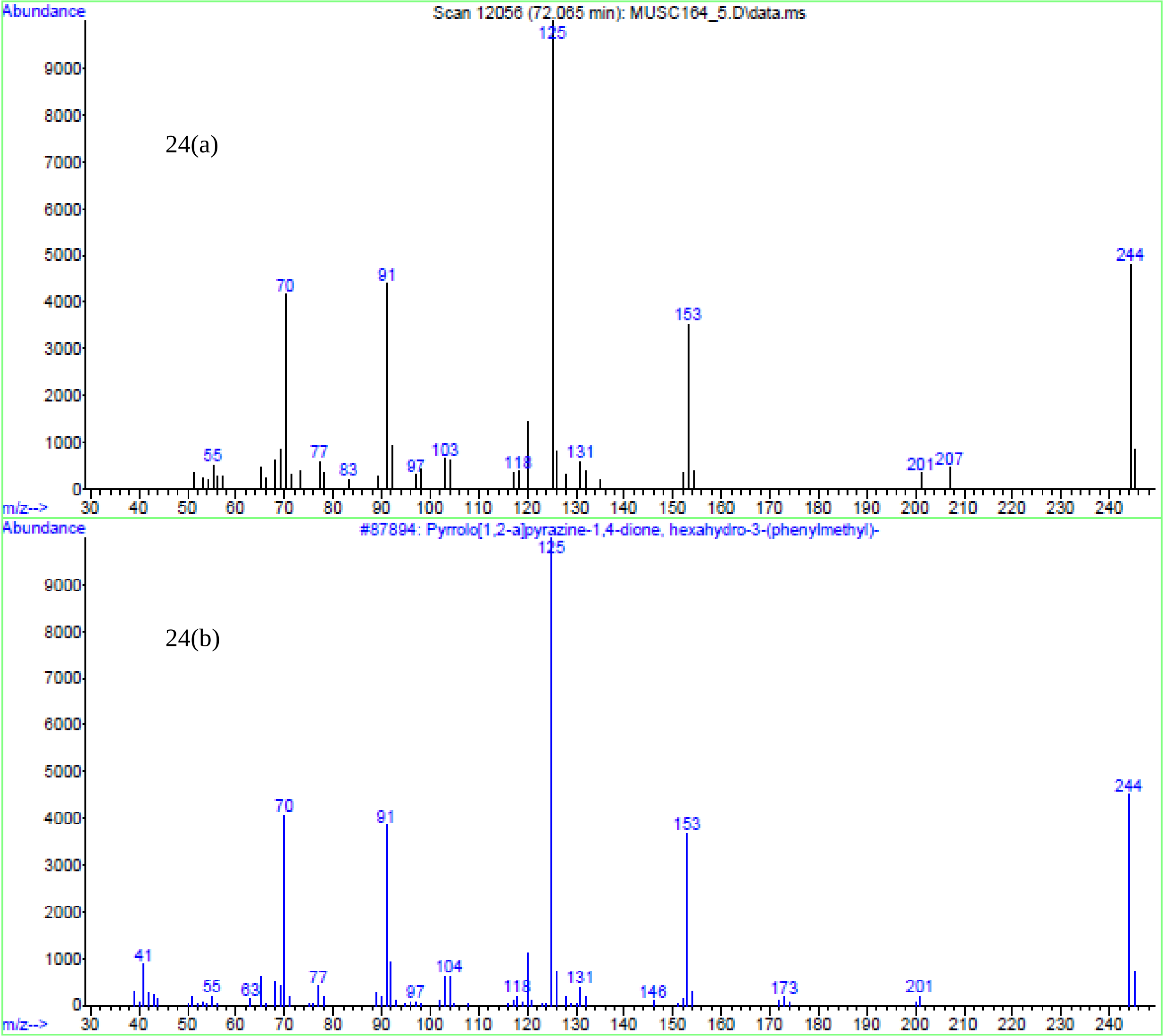

Table S1. Cultural characteristics of strain MUSC 164^T on different media at 28 ^{0}C after 7-14 days of incubation.

-, Not detected

Medium	Growth	Colony color	
		**Aerial mycelium**	**Substrate mycelium**
Yeast malt agar (ISP 2)	Good	Yellowish white	Brilliant greenish yellow
Oat Meal agar (ISP 3)	Good	Yellowish Gray	Pale yellow
Inorganic Salt Starch agar (ISP 4)	No growth	-	-
Glycerol Asparagine Agar Base (ISP 5)	Good	Yellowish white	Pale greenish yellow
Peptone Yeast Extract Iron agar (ISP 6)	Good	Pale yellow	Vivid greenish yellow
Tyrosine agar base (ISP 7)	Good	Yellowish white	Pale greenish yellow
Streptomyces agar	Moderate	Pale yellow	Grayish yellow
Starch casein agar	Good	Yellowish white	Pale orange yellow
Actinomycete isolation agar	Good	Pale greenish yellow	Yellowish white
Nutrient agar	Good	Yellowish white	Brilliant greenish yellow
